# Two-step extinction of Late Cretaceous marine vertebrates in northern Gulf of Mexico prolonged biodiversity loss prior to the Chicxulub impact

**DOI:** 10.1038/s41598-020-61089-w

**Published:** 2020-03-06

**Authors:** Takehito Ikejiri, YueHan Lu, Bo Zhang

**Affiliations:** 1 Alabama Museum of Natural History, University of Alabama, Tuscaloosa, AL 35487 USA; 2 Department of Geological Sciences, University of Alabama, Tuscaloosa, AL 35487 USA

**Keywords:** Palaeoecology, Palaeontology, Palaeoecology

## Abstract

Recent studies on mass extinctions are often based on the global fossil record, but data from selected paleogeographic regions under a relatively constant paleoenvironmental setting can also provide important information. Eighty-nine marine vertebrate species, including cartilaginous and bony fish and marine reptiles, from northern Gulf of Mexico – located about 500 km from the Chicxulub crater – offer a unique opportunity to determine an extinction process during the last 20 million years of the Late Cretaceous. Our diversity data show two separate extinction events: (i) the ‘Middle Campanian Crisis’ (about 77 Mya) and (ii) the end-Maastrichtian (66 Mya) events. Whether this stepwise pattern of extinctions occurred locally or globally cannot be determined at present due to the lack of a dataset of the marine vertebrate record for reliable comparison. However, this stepwise pattern including the Middle Campanian and end-Maastrichtian events for, at least, a 13 million-year interval indicates long-term global marine environmental changes (e.g., regression, ocean water chemistry change). Because most Cretaceous marine vertebrates already disappeared in the Gulf of Mexico prior to the latest Maastrichtian, the Chicxulub Impact may not be considered as the most devastating extinction event for the community.

## Introduction

The end-Cretaceous mass extinction event has been intriguing many researchers for decades as one of the most fascinating topics in Earth’s history^1,2^, but the main cause of this devastating incident is still under hot debate. Several competing hypothetical scenarios have been regularly studied, including large bolide impacts (e.g., the Chicxulub), extensive volcanisms (the Decan Trap), global sea-level changes, and so on. This ambiguity often comes from types of data used to quantify and determine extinction patterns, besides a complex nature of the process. Also, types of data, such as global (strictly based on a broad geologic time scale) or local (a selected geographic region in ecologic time), may provide a different view of mass extinctions^3^. The latter type, the bottom-up approach, can be specifically important for filling missing pieces of a puzzle for an overview of a mass extinction event, besides the global data-based top-down approach.

The top-down approach based on global data tends to have been popular for mass extinction studies of Mesozoic marine vertebrate^4–8^; however, data from a specific region is generally scarce in the literature^9–11^. In contrast to marine vertebrates, extinction patterns have been documented well in marine invertebrate and plankton taxa using the bottom-up approach, such as layer- or strata-level occurrence in scoped geographic regions. This tendency of taxonomic preference for mass extinction studies raises the question of whether marine vertebrates exhibit a different extinction pathway when compared to non-vertebrate marine taxa, possibly, due to unique ecological habitats (e.g., tiering, motility, feeding mechanism^3^), paleogeographic distributions, and/or species longevity.

**Figure 1:**
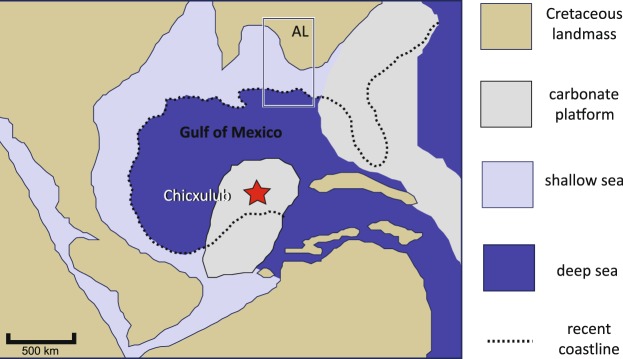
Paleogeographic map of the Gulf of Mexico about 66 million years ago. The red star indicates the position of the Chicxulub impact site. The current position of Alabama (AL) denoted by the white box is approximately 500 km from the impact site. The Mississippian Embayment is located on the left side of the symbol AL. The map was modified from Scotese^82^.

We present overall extinction patterns of Late Cretaceous marine vertebrates (cartilaginous fish, bony fish, and marine reptiles) from northern Gulf of Mexico primarily following a preliminary study^12^. This study focuses on the fossil record from northern Gulf of Mexico (the current location of Alabama in the Southeastern U.S.A) (Fig. 1). This narrowly selected geographic region can be important for marine vertebrate extinctions in the following aspects. First, successive geologic units of an over 20 million-year interval of the latest Cretaceous exist in the area (Supplementary Fig. S1). Those strata allow investigating the long-term diversity and extinction processes. Second, the region was paleoenvironmentally consistent to some degree (i.e., offshore marine environment near the Mississippian Embayment along with the southern coast of the Appalachia landmass^13^). Third, Alabama has a long history of scientific investigations and systematic fossil collecting since the early 19th Century^14,15^. This effort leads to a tremendous amount of fossil specimens, which makes it possible to apply the bottom-up approach to understanding extinction patterns. Lastly, Alabama has located about 500 km from the Chicxulub impact site in the Cretaceous Gulf of Mexico. This physical distance is paleogeographically intriguing when determining a magnitude of the asteroid impact on the marine vertebrate fossil record through the K-Pg boundary (Supplementary Fig. S2).

To quantify diversity and extinction patterns of Cretaceous marine vertebrates, species counts, percentages, and three types of rates are compared in five-time bins (stratigraphy-based units) over a 20 million year-interval (Table 1). Species occurrences including and excluding singletons were analyzed separately for comparisons. Data are analyzed on not only all marine vertebrates but also three finer taxonomic groups (cartilaginous and bony fish and marine reptiles) and some selected key Cretaceous taxa (family or order levels) to determine extinction selectivity. Moreover, other major extinction events, besides the end-Maastrichtian event, are investigated in various taxonomic groups. Following those themes based on the local data, we will discuss the possibility of the global phenomenon for marine vertebrates and other marine taxa (invertebrates and plankton) and a possible cause(s) of extinction events.

**Table 1:** List of five stratigraphic units (used as time bins for this study) based on Upper Cretaceous geologic units (formations and members) in Alabama.

Stratigraphic units	Age	Geologic units	Genus & species counts^a^	Specimen (all)^a^	counts (w/ taxonomic ID)
Unit 5	upper Maastrichtian	Prairie Bluff Chalk Fm Providence Sandstone Fm	12 gen., 16 spp. 1 gen., 1 sp.	203 3	96 –
Unit 4	lower Maastrichtian	Ripley Fm	4 gen., 9 spp.	139	37
Unit 3	middle to upper Campanian	Demopolis Chalk Fm	10 gen., 23 spp.	211	63
Unit 3	lower Maastrichtian	Bluffport Marl Mbr*	1 gen., 1 sp.	40	—
Unit 3	moddle to upper Campanian	Cusseta Sand Mbr**	3 gen., 3 spp.	9	—
Unit 3	middle Campanian	Arcola Limestone Mbr***	5 gen., 5 spp.	21	—
Unit 2	lower to middle Campanian	Mooreville Chalk Fm	33 gen., 66 spp.	6,147	1980
Unit 2	lower to middle Campanian	Blufftown Fm		216	—
Unit 1	upper Santonian	Eutaw Fm	12 gen., 49 spp.	943	461

Species and genus counts and rock volume of each geologic unit (based on surface area and volume) are compared. Key lithological features are listed in Supplementary Table S1. Data for taxonomic counts are available in Supplementary Table S3.

^a^Including specimens with uncertain taxonomic identification (data updated from Ikejiri *et al*.^12^).

*A part of the Demopolis Chalk Fm. **A part of the Reply Fm. ***A part of the Mooreville Chalk Fm.

## Results

**Figure 2:**
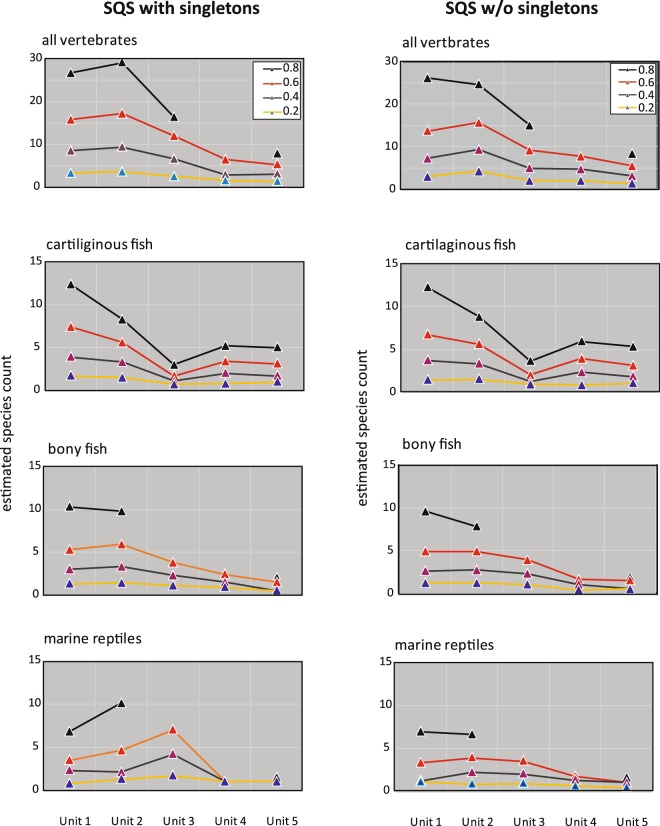
Subsample-level diversity of Late Cretaceous marine vertebrates from northern Gulf of Mexico. **Left**: including all taxa; **right**: excluding singletons. Standardized genus diversity is based on the shareholder quorum subsampling method by Alroy^17,76^. The quorum was set at 0.8, 0.6, 0.4, and 0.2 with 1,000 trials.

An overview of 8,275 Cretaceous marine vertebrate specimens from Alabama is available in Ikejiri *et al*.^12^. Stratigraphic and geographic setting (Table 1; Supplementary Fig. S1) and relative taxonomic compositions based on specimen counts (Supplementary Tables S3 and S4) were first summarized. All 8,275 specimens came from 13 counties of central to western Alabama (surface area: approximately 160 × 50 km^2^). They are housed at 12 institutions (listed in Supplementary Section 4). Of the 8,275 specimens, 3,301 specimens allowed the species-level identification with reliable stratigraphic information for this study. The sampling strategy (Table 2) and relative species richness based on rarefaction curves (Supplementary Fig. S3) and the Shareholder Quorum Subsampling (Fig. 2; Supplementary Table S2) are discussed below.

**Table 2:** Sampling variation of Cretaceous vertebrate fossils from Alabama.

Raw data						
	County	Locality	Surface area^a^	Thickness maximum^b,c^	Thickness median^b,c^	Duration (median)^d^
	(#)	(#)	(km^2^)	(m)	(m)	(m.y.)
Unit 1	12	19	4539	61	46	3
Unit 2	17	79	3978	183	96	5
Unit 3	7	39	3168	151	140	8
Unit 4	8	21	2045	76	43	2
Unit 5	7	28	1884	91	59	4
**Kendall’s tau correlation**						
	**County**	**Locality**	**Surface area**	**Thick-max**	**Thick-median**	**Duration**
County		0.796	0.197	0.796	0.796	0.796
Locality	−0.105		1.000	0.014	0.142	0.142
Surface area	0.527	0.001*		1.000	1.000	1.000
Thickness (max)	−0.105	1.000	0.001*		0.142	0.142
Thickness (median)	−0.105	0.600	0.001*	0.600		0.014
Duration	−0.105	0.600	0.001*	0.600	1.000	

**Top**: Raw data of county numbers, Locality numbers, rock volume parameters (area and thickness), and duration. The duration is estimated based on the median of an approximate unit interval for each stratigraphic unit (Supplementary Fig. S1 left). **Bottom**: Results of Kendall’s tau correlation. The numbers above the diagonal are the τ values, and the numbers below the diagonal are the p*-*values. An asterisk mark indicates a strongly correlated value.

Data sources: ^a^Based on a 1:250,000 state map. ^b^Based on Raymond *et al*.^65^. ^c^Soller (1995). ^d^Supplimentary Fig. S1.

In total, 71 genera and 89 species of marine vertebrates were identified, including 17 uncertain species-level identification, from the five stratigraphic units: Unit 1 (lower Santonian) to Unit 5 (upper Maastrichtian) ranging from 86 to 66 million years ago (Supplementary Tables S3 and S4). Those Cretaceous marine vertebrates include 26 genera and 38 species of cartilaginous fishes (sharks, rays, and chimeras), 20 genera and 24 species of bony fishes (actinopterygians and a sarcopterygian), and 21 genera and 28 species of marine reptiles (mosasaurs, plesiosaurs, and sea turtles). Of the 89 species, 28 taxa represent a singleton status (i.e., 30.8% of the total species count) including 12 cartilaginous fish, five bony fish, and 11 reptilian species.

**Figure 3:**
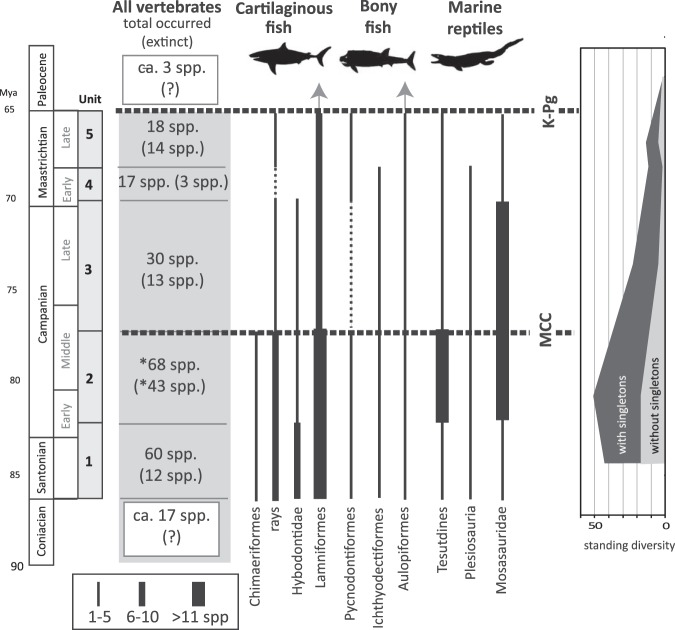
Biostratigraphic occurrence and diversity of Late Cretaceous marine vertebrates from northern Gulf of Mexico. Data show two major extinction events: Middle Campanian Crisis (MCC) and end-Maastrichtian (K–Pg) events. Standing diversity is calculated separately based on species counts with and without singletons.

In the raw data with Lazarus occurrences, 89 species occurred 193 times (and 62 species with 155 occurrences in the data without singletons) in the five stratigraphic units. Of the five stratigraphic units, Unit 2 had the largest number of occurrences (n = 68 including singletons; n = 51 excluding singletons) (Supplementary Table S5). Those data indicate that the Early to Middle Campanian interval (Unit 2) represents the diversity peak of those marine vertebrates in northern Gulf of Mexico (Fig. 3). The least number of occurrences (n = 17 in the data with singletons) was found in Unit 4 (lower Maastrichtian). The Unit 5 (middle to upper Maastrichtian) also showed a considerably low number (n = 15 in the data without singletons). Those small numbers indicate that the diversity level was constantly low in the nearly entire Maastrichtian (Unit 4 and Unit 5). A declining diversity pattern appeared in Unit 3 to Unit 4 in the all vertebrate group and each of three subgroups, cartilaginous fish, bony fish, and marine reptiles.

**Figure 4:**
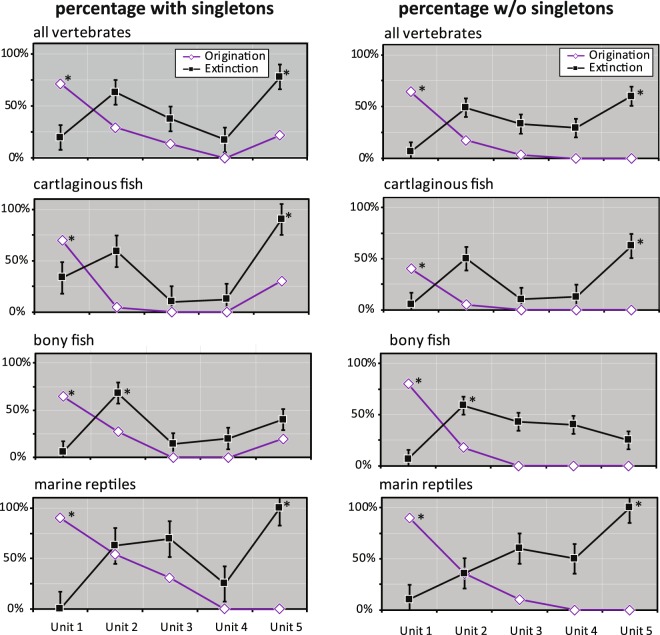
Origination (O) and extinction (E) percentages of Late Cretaceous marine vertebrates from northern Gulf of Mexico. Left: including all taxa; right: excluding singletons. The data used for this figure are listed in Supplementary Table S5. An asterisk mark indicates a significantly high percentage based on the upper 95 CI. Error bars of extinction percentage represent 95% confident intervals (following refs. ^80,83^).

Origination percentages were calculated in each time bin. The largest origination value occurred in Unit 1 (upper-most Santonian to lower Campanian) for all marine vertebrates in both types of the datasets with and without singletons (71.7% and 64.4% respectively) (Fig. 4; Supplementary Table S5). The origination percentages rapidly decreased at Unit 2 and maintained considerably low values from Unit 3 to Unit 5 as seen in the species count data. No marine vertebrate species originated (0.0%) in Unit 4. Those origination data suggest that diversity has remained noticeably low in through the nearly entire Maastrichtian for an approximately 8 million-year duration in this paleogeographic region.

Both including and excluding singleton data sets showed significantly high extinct species counts (higher than the upper 95% CIs) in Unit 2 for all vertebrates and the three subgroups (except for the marine reptiles without singletons setting) (Table 3; Supplementary Table S5). In the data with singletons, 40 marine vertebrate species disappeared while 25 was counted in the data without singletons. In all marine vertebrates, Unit 5 representing the latest Maastrichtian interval exhibited the second largest number of extinct species count. Each of the three subgroups, however, showed a slightly variable pattern of the count across the stratigraphic units. Overall, cartilaginous fish showed a considerably high number in Unit 5 (n = 9 with singletons; n = 5 without singletons), but bony fish and marine reptiles had an earlier declining signal in Unit 2 and Unit 3 (by the end of the Middle Campanian and around the Campanian–Maastrichtian boundary, respectively). At first glance, the extinct species counts suggest slightly different pathways of diversity loss among the three marine vertebrate groups.

**Table 3:** Extinction selectivity for selected Late Cretaceous marine vertebrate groups from northern Gulf of Mexico.

Higher taxa	Key taxa	Specimen #s (^b^)	Extinction percentage (%)					Extinct species count
			Unit 1	Unit 2	Unit 3	Unit 4	Unit 5	
Chimaeriformes	*Edaphodon*, *Ischyodus*	26	0.0	100.0*	0.0	0.0	0.0	4
rays^a^	*Borodinopristis, Brachyrhizodus, Pseudohypoliphus*	280	66.7	80.0*	50.0	0.0	100.0*	12
Hybodontiformes + Ptycodontiformes	*Ptychodus*, hybodontids	151	71.4	80.0	100.0	NA	NA	6
Lamniformes	*Cretalamina*, *Scapanorhynchus, Squalicorax*	1,243	16.7	27.3	0.0	0.0	85.7	13
Aulopiformes	*Enchodus*, *Stradodus*	857	0.0	33.3	25.0	25.0	33.3	3
Ichthyodectiformes	*Ichthyodectes*, *Xiphactinus*	244	0.0	50.0	50.0	100.0*	NA	4
Mosasauridae	*Clidastes*, *Mosasaurus*, *Tylosaurus*	1,563	0.0	63.6	57.1	0.0	100.0*	12
Testudines	*Ctenochelys, Protostega, Toxochelys*	1,250	0.0	54.5	60.0	50.0	100.0*	11
Plesiosauria	polycotylid sp., elasmosaurid sp.	56	0.0	33.3	50.0	100.0	NA	2

Extinction percentages of raw data are compared through the five-time bins. Numbers with an asterisk mark indicates a significantly high value.

^a^Including Myliobatiformes, Orectolobiformes, Rajiformes, Sclerorhynchiformes, and Squatiniformes.

^b^Data updated from Ikejiri *et al*.^12^.

While many marine vertebrate species disappeared just before the end-Maastrichtian (Unit 5), at least, three species survived through the K–Pg contact in northern Gulf of Mexico (Fig. 3; Supplementary Table S4). Those included the genus *Enchodus* (including two species *E. ferox* and *E*. *petrosus*: Aulopiformes) and *Cretalamna* (*C*. *appendiculata*: Lamniformes). Those K-Pg survivors may be considered as Dead Clade Walking (i.e., referring to extinction debt when a few still survive after a devastating event^16^). Based on the last occurrence data in Unit 5, possible victims around the K–Pg boundary were a few species of mosasaurs and protostegid turtles. Most lineages of rays (Myliobatiformes, Orectolobiformes, and Sclerorhynchiformes) and pycnodontiform bony fish also disappeared below the K–Pg. It is, however, worth noting that the magnitude of species declines could be greater in the earlier time (Unit 2 and/or Unit 3) than in the end-Maastrichtian extinction event (Unit 5). This earlier declining pattern is particularly applied for bony fishes and marine reptiles (see also other extinction values below).

Of all marine vertebrates, the largest and significantly high extinction percentage (Materials and Methods) was found in Unit 5 representing the K-Pg extinction based on the upper 95% CI (83.3% with singletons; 60.0% without singletons) (Fig. 4; Supplementary Table S5). Unit 2 in middle Campanian also showed considerably high extinction percentages in the two datasets, but no other units showed notably high values for all vertebrates. As seen in the extinct species counts, the cartilaginous fish and marine reptiles showed significantly high values in the latest Maastrichtian (Unit 5), but bony fish did not show any signs of devastation. Notably, in Unit 2 (lower to middle Campanian), the two fish groups exhibited high-level extinction pressure in both datasets. However, marine reptiles showed a moderate (in the singleton dataset) or very low extinction level. Only marine reptiles displayed a notably high extinction percentage in Unit 3 as also found in the species counts. Those data on the extinction percentage indicate that those marine vertebrates have different extinction patterns in the Late Cretaceous and multiple extinction events might occur such as in Unit 5 (i.e., the end-Maastrichtian) and Unit 2 (the end of middle Campanian) (Fig. 3).

Some common Late Cretaceous marine vertebrate taxa tend to have followed this overall extinction pathway – a combination of two large extinction impulses in Unit 2 (middle Campanian decline) and Unit 5 (late Maastrichtian to the K-Pg boundary). Those taxa specifically include chimeras, rays, hybodontid sharks (including Hybodontiforms and Ptychodontiformes), aulopiform fish, ichthyodectiform fish, and mosasaurs, based on extinction percentages (Table 3). Some other fish taxa, however, showed slightly different extinction pathways. For example, lamniform sharks showed a moderate-level extinction percentage in Unit 2. Then, they survived fairly well in Unit 3–Unit 4 and until hitting the major devastation in Unit 5. The single species of hybodontiform/ptychodontiform, *Ptychodus mortni*, might survive until Unit 3, but most of hybodontid and ptycodontod species disappeared by the end of Unit 2.

Different extinction patterns were also identified in the three marine reptiles, mosasaurs, sea turtles, and plesiosaurs. Many of those reptiles commonly exhibited a strong late Campanian declining trend (Unit 2 and Unit 3) based on a number of extinct species and the extinction percentages (Fig. 3; Table 3). In sea turtles including bothremydids, stem-basal chelonioids, and protostegids, while the highest extinction percentage appeared in Unit 2, they tend to have decreased continuously from Unit 2 to Unit 5. Plesiosaurs showed a very scatter fossil record from Alabama including an indeterminate elasmosaurid and polycotyrid taxa; Supplementary Table S3). The last occurrence of plesiosaurs is Unit 4, but no record of Unit 5 has been known. Mosasaurs have the 100% extinction rate at the K-Pg boundary but include only two species *Mosasaurus maximus* (cf. *M. hoffmani*) and *Plioplatecarpus* sp. in Unit 5. The highest number of mosasaurs (n = 11) disappeared in middle Campanian (11 species in Unit 2 consisting of 63.3%), and this declining trend followed in later Campanian (57.1% in Unit 3).

**Figure 5:**
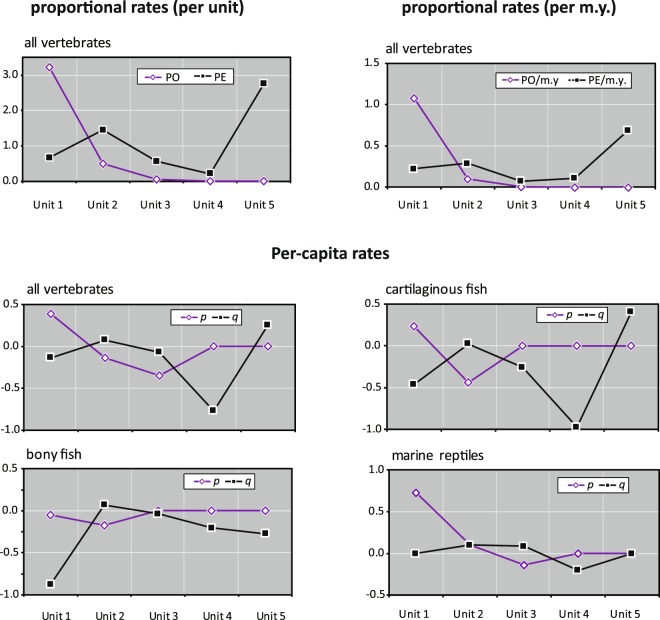
Origination and extinction rates of Late Cretaceous marine vertebrates from northern Gulf of Mexico. Three types of rates are compared based on data without singletons, including PO (proportional origination), PE (proportional extinction), p (per-capita origination), and q (per-capita extinction) (see Materials and Methods). The original data and other kinds of extinction rates are available in Supplementary Table S6.

Three types of extinction rates, proportional extinction (PE), proportional extinction rate per million years (PE m.y.), and per-capita extinction rate (*q*), were calculated solely based on the data excluding singletons (Materials and Methods). The two latter rates incorporate data of a duration of a time interval (stratigraphic unit) while the first one does not. In our dataset, the five stratigraphic units have a different duration ranging from approximately 2 to 8 million years (Table 2; Supplementary Fig. S1). Overall, the three types of rates of all vertebrates (Supplementary Table S6) showed a similar overall extinction pathway (i.e., a two-step diversity decline process in Unit 2 and Unit 5) as seen in the species count and extinction percentage (Fig. 4). In per-capita extinction rates (Fig. 5), the latest Maastrichtian (Unit 5) has the highest value, which is mainly based on cartilaginous fish. The highest value was also identified in Unit 2 for the all vertebrate and the two fish groups.

## Discussion

### Sampling effects and diversity comparisons

Diversity analyses for the fossil record cannot completely avoid the possibility of data distortion due to inconsistent fossil collecting (sampling), various conditions of preservation, different sedimentological settings, and so on^17^. To determine the risk of those kinds of potential biases, sampling variations and estimated species numbers generally provide some intuitions. In this study, six parameters of sampling variations in the duration in million years, fossil sites, and rock volume are compared (Table 2). Among those parameters, the surface area of each Formation (or Member) has the strongest tendency of correlation, especially, with the duration and relative strata thickness in the dataset. The numbers of localities (fossil sites) and counties (in Alabama) may contain a possible limitation (i.e., the largest number of fossil sites assigned to Unit 2), but other units tend to be constant.

Regarding relative species richness, the rarefaction curves of all vertebrates and the three sub-vertebrate groups show a reasonably robust sample size in our dataset (Supplementary Figs. S3). The Shareholder Quorum Subsampling (SQS) at four different quora share similar overall topology of the diversity curve in all vertebrate and each of the three sub-groups (Fig. 2) (Supplementary Table S2). The highest diversity appears in Unit 2 and a continuous decline in Unit 3 to Unit 5 for all vertebrates. The three vertebrate subgroups, however, show slightly different patterns in the SQS curves. The evident difference appears in marine reptiles that exhibit the diversity peak at Unit 3. Cartilaginous and bony fish groups show similar diversity trends in overall, but a sharper decline occurs from Unit 2 to Unit 3 in the former group. Those different pathways of the three vertebrate subgroups reflect the real diversity pattern in our dataset. Otherwise, if our data are heavily distorted by different sampling strategies or preservational settings, all three groups will likely show the same pattern. In sum, we conclude that the data from the 3,301 vertebrate specimens is robust enough for further discussion of extinction patterns and processes.

### With or without singleton taxa

Including or excluding singletons taxa has been an important issue for diversity analyses in the fossil record^18,19^. While many studies exclusively exclude singletons, some argue possible advantages for using taxa occurred in a single interval^20^. The extinction percentages of our marine vertebrate data show an overall similar extinction trend in both datasets with and without singletons (Fig. 4). However, the proportional and per-capita extinction rates of all vertebrates that exclude all singleton counts display a few notable differences among the three sub-vertebrate groups (Fig. 5; Supplementary Table S6). For example, marine reptiles have much smaller extinction rates in the latest Maastrichtian (Unit 5) than in the Campanian (Unit 2 and Unit 3). Also, bony fish does not show a decline signal in Unit 5 based on all extinction rates. Those patterns are largely not observed or not evident in the extinction percentages with the singleton data (Fig. 4).

We, thus, suggest that excluding singletons from our dataset possibly hide some important extinction signals or, at least, do not provide fine resolution to interpret the extinction trend. One of the reasons for the possible singleton effect is due to a relatively small number of time units (e.g., losing all bottom-boundary crossing taxa in Unit 1 when excluding singletons). Furthermore, some singleton taxa (listed in Supplementary Table S3) excluded in the all extinction rate analyses have a (relatively) high number of specimens^12^. This fact indicates that some or most singleton taxa in our dataset most likely represent a true diversity pattern (i.e., single time occurrence). Theoretically, finer biostratigraphic data from subdivided geologic units (e.g., Formation, Member) or strata-level occurrence can reduce a total singleton count in the dataset. This kind of approach must provide a better resolution of the extinction pattern although it is not practical at this moment. Therefore, we think that incorporating the two types of datasets is necessary for those marine vertebrates.

### How many extinction events?

While 88 out of 90 marine vertebrate species became extinct for an over 20 million-year interval of the latest Cretaceous, two considerably large extinction events are recognized based on the data with singletons (Fig. 3). The largest extinction magnitude in all marine vertebrates is identified in Unit 5, which represents the end-Maastrichtian extinction event. Although extinct species counts are considerably low in Unit 5 (Supplementary Table S5), this extinction event had certainly impacted the marine vertebrate community near northern Gulf of Mexico. Of the three vertebrate groups, cartilaginous fish displays the severest devastation (Figs. 4 and 5). Bony fish and marine reptiles, however, do not show a strong signal of diversity loss. Different extinction pathways in the three vertebrate groups indicate a possible complex process with different causes toward the end-Maastrichtian.

Another large extinction event is identified in Unit 2 during the Middle Campanian (Fig. 3). This ‘Middle Campanian Crisis’ event is characterized by a combination of significantly high diversity and a sharp decline in the time interval (Figs. 4 and 5). The two fish groups tend to be involved more explicitly than marine reptiles. In particular, bony fish has the largest extinction magnitude through the five Late Cretaceous units. In marine reptiles, some species also disappeared during the Middle Campanian Crisis, but the majority of mosasaurs, plesiosaurs, and sea turtles have vanished in the Late Campanian to the earliest Maastrichtian (Unit 3) in northern Gulf of Mexico.

Many studies on marine vertebrate extinctions have emphasized the end-Maastrichtian event (e.g., marine reptiles^4,6,8^, mosasaurs^7^, plesiosaurs^21^, sharks^11^, bony fish^5^) while a few studies have also pointed out the possibility of Campanian extinctions (e.g., actinopterygian and mosasaur fauna in Sweden^22,23^). Our study suggests that species-level data from a selected geographic region have some advantages to reveal the Middle Campanian biodiversity loss. In contrast to Cretaceous marine vertebrates, some studies of marine invertebrates and plankton show signals of a large extinction magnitude that can be referred to as the Middle Campanian Crisis. For example, some mollusks show evident declined patterns in the Middle to Late Campanian (e.g., ammonites^24–28^, gastropods^29^, inoceramids^30,31^, rudists^32^, a combination of various taxa^33,34^). In marine plankton, some studies display continuous background extinctions throughout the Campanian (e.g., nannoplankton^35,36^, foraminifera^37,38^).

Near the northern Gulf of Mexico region, detailed extinction patterns have not been well known for most Cretaceous marine taxa. A few previous studies on mollusks^39^ and plankton^40^ cover only selected layers of the upper-most Maastrichtian formations (i.e., the upper part of Unit 5), but no published data are available for the Campanian and early Maastrichtian records. Hypothetically, non-vertebrate marine taxa may have a different extinction pathway from marine vertebrates since due to various types of paleoecological (e.g., life habitats and modes, relative trophic level positions) and biological factors (e.g., species longevity, body size)^41,42^. To further investigate this hypothetical scenario, data of strata- or layer-based fossil occurrence for selected taxa will be necessary.

### Local vs. global phenomena?

Could this Middle Campanian Crisis be paleogeographically a global phenomenon for the marine ecosystem? To date, no comprehensive data to outline spatial extinction patterns of all marine vertebrates are available in the literature. We have attempted to investigate the Middle Campanian Crisis in global-scale data of marine vertebrates in the Paleobiology Database (Supplementary Tables S7 and S8). As for a reference, a total of 396 genera of marine vertebrates recorded from five intervals, using an 8 million-year time bin for each, from the Cenomanian to the end of the Paleocene (about 40.1 million years in total duration) occur 690 times in total. The genus-level based global data show the largest extinction percentage (57.2%: Supplementary Table S7) at the latest Cretaceous time bin for all vertebrates, cartilaginous fish, and marine reptiles.

There are difficulties to draw a clear conclusion of whether the Middle Campanian Crisis involved marine vertebrates on a global scale. The main reason is that many taxa in the dataset exhibit uncertainty in alpha taxonomy at the species-level identification and even in higher-levels (e.g., family, order). Those include some major or relatively common Cretaceous marine vertebrate taxa, specifically assigned to rays, lamniforms, crossognathiforms, ichthyodectiforms, tselfatiforms, and sea turtles. Another challenge in using global data lies in the limitation of the stratigraphic setting. The database does not provide robust data to extract a time interval that matches the Middle Campanian for quantitative comparisons with our data. Thus, we suggest that the global data of Cretaceous marine vertebrates presented here is a reference for general information and further analysis of the global data for detailed diversity patterns is needed (currently under study by one of the authors, T. I.).

### Potential cause(s) of the middle campanian crisis

Of the two extinction events of Late Cretaceous marine vertebrates in northern Gulf of Mexico, the Chicxulub impact is likely the strongest candidate for the main cause of the latest Maastrichtian devastation^43,44^ (Supplementary Fig. S2). Many studies reveal a series of aftermath global marine environmental changes triggered by the impact, such as impact bursts^45^, mega-tsunami^46,47^, and climate changes^48–51^. Since Alabama is physically located merely 500 km away from the impact site (Fig. 1), this catastrophic event likely affected the 12 species that disappeared during the time of Unit 5, and as the result, iconic Cretaceous marine vertebrates, mosasaurs, sea turtles, a few groups of rays, and possibly lamniform sharks were completely wiped off from the Gulf of Mexico.

Determining the main physical cause(s) of the Middle Campanian Crisis is more challenging for the marine vertebrate community. To our knowledge, the globally impactful event at the corresponding time and space is uncertain. Some kinds of global long-term marine environmental changes in the Late Cretaceous, however, can be considered as possible candidates. Those include, for example, sea-level change (esp., global regression^52–55^), faunal change in plankton^40^, marine anoxia^56^, ocean acidification^49,57–59^, and the Strangelove oceans^58,60^. Among those hypotheses for a global scale, circumstantial evidence from northern Gulf of Mexico indicates a series of regression events (e.g., refs. ^61,62^) that must affect marine vertebrate diversity to some degree (Supplementary Fig. S4). Moreover, an alternative possibility is a relatively large asteroid impact in central Alabama. The Wetumpka Impact crater, exhibiting 7.6 km in diameter, is estimated to occur sometime in the time of the Mooreville Chalk (Unit 2: ca. Early to Middle Campanian)^63^. To further investigate this hypothetical scenario, more precise data on the impact age and magnitude will be needed.

## Materials and Methods

### Geologic setting

Following Ikejiri *et al*. (ref. ^12^), Upper Cretaceous geologic units (a combination of formations and members) were subgrouped into five successive stratigraphic units (Table 1; Supplementary Table S1). Surface rocks of those Cretaceous units are geographically distributed in the mid-region from northwestern to central-eastern Alabama (Supplementary Fig. S1). Surface area data of each unit are available in the USGS Geologic maps of US states (ref. ^64^ accessed on July 2016). Ages of the geologic formations and members are based on ref. ^65^ and USGS Geolex^66^. Using a Formation- and Member-based time setting can provide finer intervals than numerical values (e.g., 10 million-year) when determining extinction and diversity patterns^67^. The five successive units used in this study exhibited approximately 20 million- year total duration, which consists of about a 4 million-year bin for each unit. Most of the marine vertebrate fossils from Alabama do not have layer- or strata-level stratigraphic information.

In Alabama, an unconformity might occur twice in the upper Cretaceous units: in the contact of the Prairie Bluff Chalk (upper Maastrichtian) – the Clayton Formation (lower Paleogene) and within the Reply Formation (lower Maastrichtian). Those unconformities can be arguable and may occur only regionally (e.g., refs. ^13,68^). In the K–Pg contact between the upper Maastrichtian Prairie Bluff Chalk and the Paleogene Clayton Formation (Supplementary Fig. S2), nannoplankton data indicate a regional unconformity ranging from 0.4 million to possibly over a few million years^69–71^. Strontium isotope and paleomagnetism, however, suggests a successive K–Pg boundary with no unconformity^39,72^. Possible tsunami deposits with direct impact materials (e.g., impact ejecta, glass spherules, microtektites) have been reported from several K–Pg sites near the Mississippi Embayment^71^. During a series of field investigations, we found typical Late Cretaceous taxa, such as the lamniform shark (*Squalicorax*) and mosasaur (cf. *Mosasaurus*), from the base of the Paleogene Clayton Formation (Supplementary Fig. S2 and Table S4). These data may represent a reworked condition (as suggested by refs. ^61,62^) although further investigation seems to be needed for verification.

### Sampling variations and subsampling

For sampling variations (following refs. ^73,74^), we used a correlation test to compare the relation of six sampling measures, such as (1) counties, (2) fossil localities, (3) the surface area of each geologic unit, (4) maximum and (5) median of each unit, and (6) a duration (my) for each stratigraphic unit. We used Kendall’s tau due to expecting a non-linear relation in the dataset. The PAST (version 2.08^75^)was used to run rarefaction analysis. Relative fossil richness was estimated by the Shareholder Quorum Subsampling; the quorum, µ, was set as 0.2, 0.4, and 0.8 for comparisons with a total of 1000 subsampling trials for each dataset (using the R code provided by ref. ^17^). For this analysis, using ‘two timmers’ species counts (N_2t_; ref. ^76^) was applied for specimens with reliable species-level identification when genera consist of multiple taxa. The result is shown in Fig. 3.

### Marine vertebrate fossils

Data on species counts were collected only from museum specimens that are officially curated (by the summer of 2015). Twelve institutions in the U.S. and U.K. store those specimens (Supplementary Materials Section 4). In total, over 8,275 specimens were stored in the institutions, and only ones with reliable generic level identification with valid stratigraphic information (n = 6,352) were selected for this study (Supplementary Table S3). The taxonomic status was checked mostly in actual specimens by the author (T.I.), and some results were reported^11^. The 6,352 specimens include a mix of specimens with skeletons and isolated bones that exhibited enough proportions to examine certain morphologies. Of Cretaceous vertebrate fossils from Alabama, only fully aquatic forms were scoped in this study, including cartilaginous fishes (sharks, rays, sawfish, and chimeras), bony fishes (actinopterygians and sarcopterygian fish), and marine reptiles (mosasauroid squamates, plesiosaur sauropterygians, and chelonioid testudines). Semiaquatic and fully terrestrial archosaurs, such as crocodilians, pterosaurs, non-avian dinosaurs, and birds, were not included (those excluded taxa are listed in ref. ^12^. Only specimens with bony tissues, such as skeletons, bones, and teeth, were analyzed, but scale-specimens for some fish taxa (e.g., refs. ^77,78^) were not included.

Global data of Late Cretaceous marine vertebrates were downloaded from the Paleobiology Database^79^ (http://fossilworks.org; accessed in August 2019). Stratigraphic and geographic occurrences were chosen for quantitative comparisons at the genus-level because species-level taxonomic assignments and occurrences may contain more uncertainties.

### Data quantification for extinction patterns

Supplementary Tablesbiting a singleton status (i.e., species occurred only in a single geologic unit) can yield a large amount of important information to assess extinction patterns and processes as suggested by two studies^80,81^, and those taxa were, thus, included for this study. However, data excluding singletons were also analyzed for comparison. Since there is a hiatus in the earlier Santonian (below Unit 1) in Alabama, occurrence of some species in Unit 0 (Supplementary Table S3) were based on the record from other areas of the Gulf of Mexico or the Western Interior Seaway Lazarus taxa that occurred 22 times in 13 species (seven times in Unit 3 and nine in Unit 4, and once in Unit 5) were included for all data analyses. For calculating origination (O) and extinction (E) percentages, total species counts (N) per time bin (Stratigraphic Unit) were used as O/N and E/N for the data set with and without singletons. Various extinction and origination rates with boundary-crossing measures such as (1) proportional (PE and PO), (2) proportional rate per m.y., and (3) per-capita rates (p and q), analyzed for this study followed refs. ^18,19^.

## Supplementary information

Supplementary material.
