# Supplementary material for: Two-step extinction of Late Cretaceous marine vertebrates in northern Gulf of Mexico prolonged biodiversity loss prior to the Chicxulub impact
Source: Sci Rep. 2020 Mar 6;10:4169. doi: 10.1038/s41598-020-61089-w (PMC7060338; doi:10.1038/s41598-020-61089-w)
Supplement: Supplementary file 1 — Supplementary material. [file 41598_2020_61089_MOESM1_ESM.docx]

Supplementary Materials for

**Two-step extinction of Late Cretaceous marine vertebrates in northern Gulf of Mexico** **prolonged biodiversity loss prior to the Chicxulub impact**

By

Takehito Ikejiri, YueHan Lu, and Bo Zhang

**This PDF format includes:**

1. Geologic Setting (Table S1, figs. S1–S2)

2. Analytical Biases (Table S2) and Species Richness Estimate Fig. S3)

3. Paleogeography (Fig. S4)

4. Alabama Marine Vertebrates (tables S3–S6)

5. Global Extinction Pattern of Cretaceous Marine Vertebrates (tables S7–S8)

6. References

**1. Geologic Setting**

An overview of the geologic setting is available in the main text (Methods and Material) and several references^1–4^.

**Table S1.** Key lithological features of Upper Cretaceous geologic units in Alabama. General features of those units are listed in the main text (Table 1).

| **Stratigraphic units** | **Geologic units** | **Key lithology ^1^** |
| --- | --- | --- |
| **Unit 5** | Prairie Bluff Chalk Fm | Bluish-gray firm sandy, fossiliferous chalk |
|  | Providence Sandstone Fm | Cross-bedded fine to coarse sand and white, dark-gray, and pale-red-purple mottled clay (upper part); dark gray laminated to thin-bedded silty clay and very fine- to fine-grained sand that is abundantly micaceous and carbonate (lower parts) |
| **Unit 4** | Ripley Fm | Light gray to pale olive massive bioturbated micaceous glauconitic fine sand, sandy calcareous clay, thin indurated fossiliferous sandstone (upper part); calcareous sandstone, sandy chalk and coarse cross-bedded sand with occasional thin limestone layers (lower part) |
| **Unit 3** | Demopolis Chalk Fm | Light gray to medium light gray fossiliferous chalk; thin marl beds (lower part). |
|  | Bluffport Marl Mbr* | Massive chalky very dark marl, very clayey chalk, calcareous clay |
|  | Cusseta Sand Mbr** | Sandy chalk and coarse cross-bedded sand with occasional thin limestone and fine gravel layers |
|  | Arcola Limestone Mbr*** | With 2 to 4 beds of light gray impure dens brittle fossiliferous limestone with softer marl inbedded |
| **Unit 2** | Mooreville Chalk Fm | Yellowish-gray to dark bluish-gray clayey compact fossiliferous chalk and chalky marl |
|  | Blufftown Fm | Mainly glauconitic calcareous fine sand; micaceous clay and marl, fossiliferous clay, gray calcareous fossiliferous sandstone, and calcareous clay and silt (locally variable) |
| **Unit 1** | Eutaw Fm | Light greenish gray fine to medium-grained well-sorted micaceous cross-bedded sand, fossiliferous and glauconitic in part, containing greenish-gray micaceous silty clay and medium gray-to-dark gray carbonaceous clay |

**^1^** Based on ref. 1.

*A part of the Demopolis Chalk Fm.

**A part of the Reply Fm.

***A part of the Mooreville Chalk Fm.


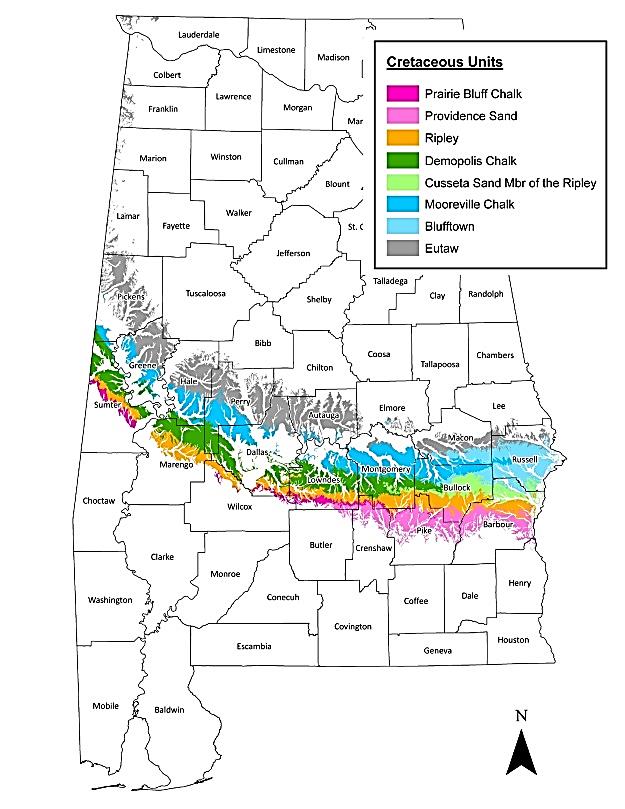

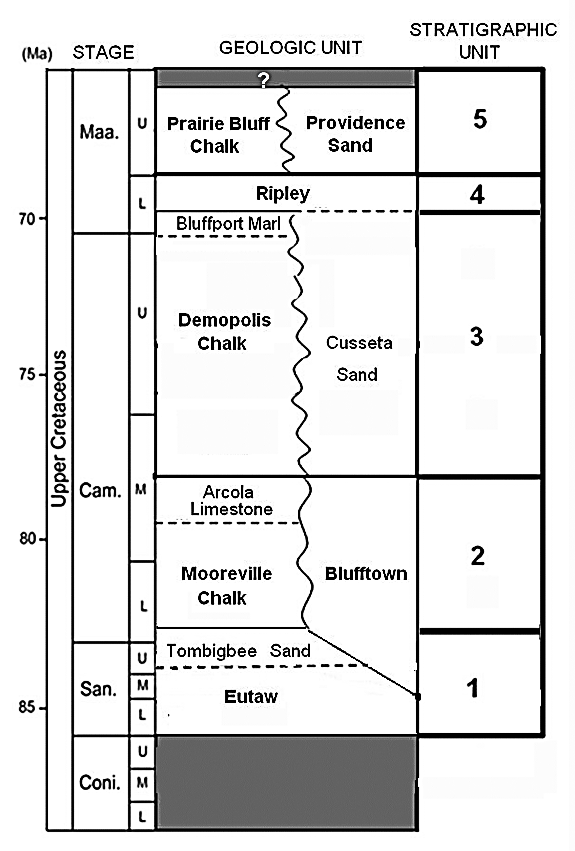


**Figure S1.** Late Cretaceous stratigraphy (left) and geologic map (right) in Alabama (modified from ref. 4).

**
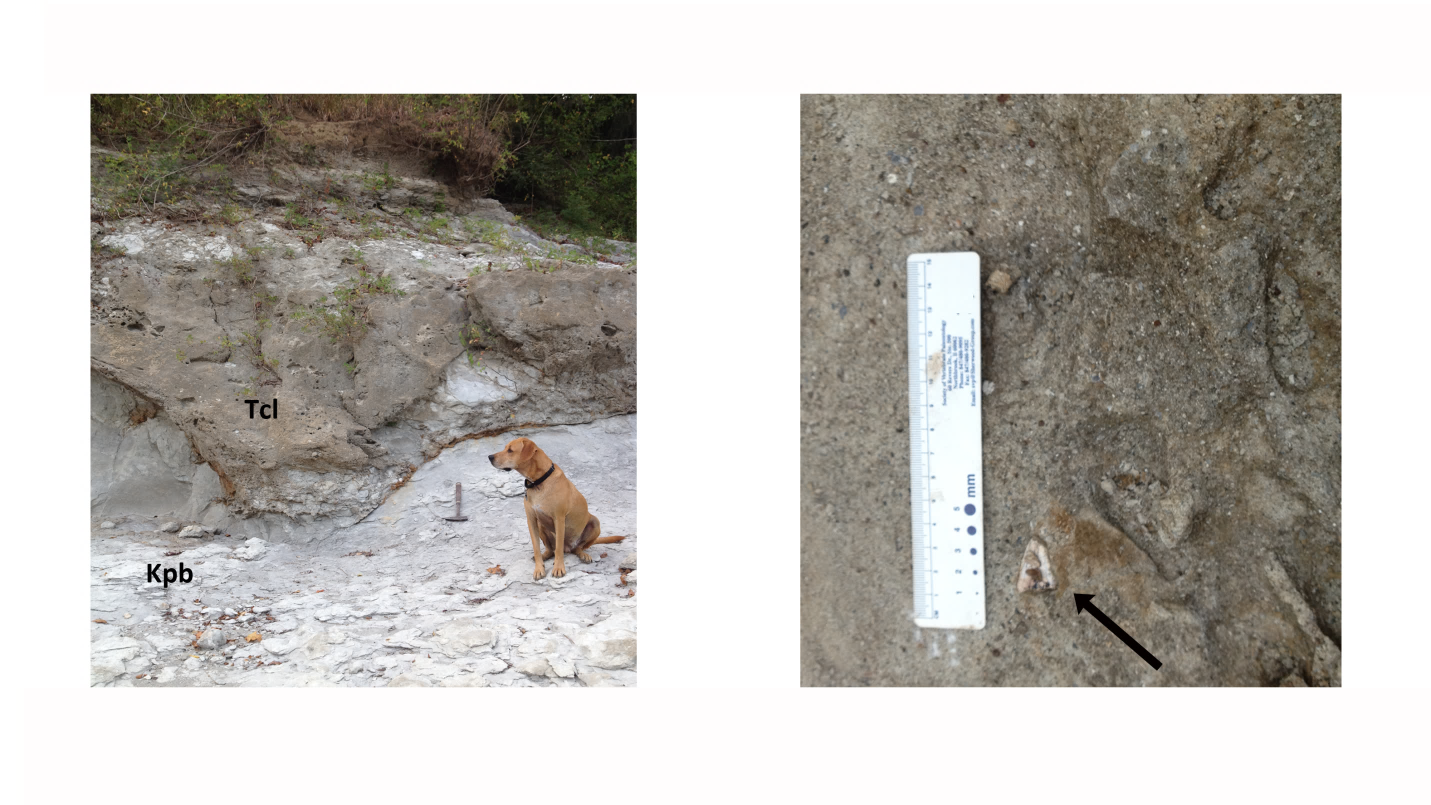
**

**Figure S2.** Moscow Landing K–Pg boundary site in Sumter County, western Alabama. **Left:** the Upper-most Cretaceous Prairie Bluff Chalk (Kpb) and the lower-most Paleocene Clayton Formation (Tcl) are shown. **Right:** a tooth of marine reptile, *Mosasaurus* sp., in the Clayton Formation. Photos were taken by one of the authors (T.I.) in summer 2015.

## 2. Analytical Biases and Species Richness Estimation

## While examining over 8,275 Cretaceous vertebrate fossil specimens (as some results presented in ref. 4), we are confident that marine vertebrate fossils have been collected thoroughly and systematically in Alabama by the 12 institutions for over 150 years. Marine reptiles and large bony fishes tend to have received special attention by field investigators, as shown in many isolated bones and even incomplete fragmentary specimens housed at the institutions. This collecting emphasis on some specific taxa (e.g., Hybodontiformes, Lamniformes, Ichthyodectiformes, Tselfatiformes, Mosasauridae, Testudinates) yield comprehensive data that reduce the risk of biases for determining extinction patterns.

## Of the total of 8,275 specimens, 3,301 specimens have species-level identification with confidence and the information of the stratigraphic unit and fossil locality for data analyses presented in this study (Table 2 in the body text). The largest number of marine vertebrate fossil specimens were collected from Unit 2 while the least number occurred in Unit 4.

## Small specimens (e.g., microscopic-sized isolated teeth) are possibly missed to be collected more often than large bones in the field as a case of sampling bias. However, a few specific fossil sites/localities along small rivers or creeks in Alabama can fill this potential gap. For example, a single fossil site along a local creek (the University of Alabama Museums locality number: AGr-43) has been producing a tremendous amount of small isolated teeth and bones that allow identifying, at least, 28 species of rays and sharks from Unit 1 and Unit 2^5^. Such microvertebrate fossil sites reduce the risk of the sampling and/or preservational bias.

Certain geologic formations or members possibly preserve vertebrate fossils better than others due to variable sedimentological and taphonomic settings. For example, Unit 2 including the Mooreville Chalk and the Blufftown Formation produces the largest number of marine vertebrate species (n=67) and specimens (n=3,978) in Alabama (Table 1) (based on ref. 4). We suggest that this large species count reflects a true diversity pattern, rather than a biased view due to a preservational or collecting bias for the following reasons. First, there is no considerably large difference in the amount of rock volume or surface areas among the Late Cretaceous units (Table 1). An exact rock volume of each geologic unit is physically difficult to measure, but a surface area (in Km^2^) and a range of thickness (in meters) allow estimating their relative sizes for quantitative comparisons. The Eutaw Formation in Unit 1 likely shows the largest rock volume, but nearly all vertebrate fossils (943 specimens) concentrate in the upper member (the Tombigbee Sand). The Prairie Bluff Chalk in Unit 5 exhibits considerably low rock volume, but a relatively large specimen number (n = 203) should provide a reasonable data size for determining the fossil abundance relative to other units.

Lithological and sedimentological features are overall consistent throughout Unit 2 to Unit 5, exhibiting mainly light-grayish calcareous chalky layers (Table S1)^1^. The only exceptional case is the Providence Sand (Unit 5), which is characterized by loose sediments (sand and clay) and distributed only in eastern Alabama to western Georgia. The formation has produced only one species and three specimens in total. The Ripley Formation (Unit 4) that is characterized by mostly calcareous sandy chalk has produced a significantly low number of vertebrate species count (n=19 spp.; 95% CI) and a relatively small specimen number (n=139), but invertebrate fossils (especially mollusks) are abundant and often well-preserved (personal observation). Mancini et al. (ref. 3) suggests a series of regression events occurred near the current location of Alabama during the early Maastrichtian. However, no considerably significant changes associated with any drastic environmental changes have been known during the time of Unit 4.

Extinction and diversity patterns are varied among subgroups of vertebrates through the time. Although bony fishes and marine reptiles have the largest degree of occurrence and extinctions in Unit 2, cartilaginous fishes show the biggest decline pattern in Unit 1 (Figs. 2 and 3 in the main text). Moreover, some smaller taxonomic groups show different timings of diversity and extinction peaks through the units (Table 2 in the main text). These variable extinction pathways across various taxonomic groups also indicate that our data represent natural phenomena of the extinction process, instead of biased views. In other words, only if a strong preservational bias is involved, the same or very similar extinction pattern would be expected to be observed across different groups. To sum, a series of circumstantial evidence indicates that our dataset is not strongly biased by sampling and fossil preservation artifacts.

Sampling variation among (1) counties, (2) fossil localities, (3) the surface area of each geologic unit, (4) maximum and (5) median of each unit, and (6) a duration (my) of each unit was investigated by a correlation test (Table 2 in the main text). Although Spearman’s rho correlation test tends to be more commonly used for this kind of analysis (e.g., refs. 6 and 7), we used Kendall’s tau due expecting a non-linear relation. Because of a small number of data entry (i.e., from the five-time units), the result may not be robust for further interpretation, but it may be a mere reference for an overview of sampling variations. Among the six parameters, the surface area shows the best nature of correlation.

**Rarefaction—**Expected species counts are calculated based on rarefaction for specimen numbers (Supplementary Fig. S3). In our dataset, a total of 8,275 specimens were collected, and we could identify at the species level for 3,301 of them. The rarefaction curve indicates our data size (i.e., 90 species in total) is reasonable for investigating a diversity analysis. The 3,301 specimens included 1,186 for chondrichthyans (38 species), 897 for bony fish (24 species), and 1,218 for marine reptiles 28 species). The rarefaction curves suggest that chondrichthyans exhibit the best reliable data set and bony fish tends to represent the weakest record.

**Figure S3.** Sample-based rarefaction curves based on the raw data of Cretaceous marine vertebrates. **Top:** all marine vertebrates; **bottom:** cartilaginous fish, bony fish, and marine reptiles. Dotted lines indicate 95% CIs.

**Subsampling analysis—**Relative fossil richness was estimated by the Shareholder Quorum Subsampling; the quorum, µ, was set at 0.8, 0.4, and 0.2 for comparisons, and a total of 1000 subsampling trials were run for each dataset (using the R code provided by ref. 8). We compared three subgroups of sample-level diversity with all vertebrates through the five geologic units. The result (Figure 2 in the main text; Supplementary Table S2) shows that the highest diversity in Unit 2.

**Table S2**. The results of the SQS of Cretaceous marine vertebrates. Data with and without singletons are analyzed separately for all vertebrates and the three sub-groups.

| **All vertebrates** | | |  | |  | |  | |  |
| --- | --- | --- | --- | --- | --- | --- | --- | --- | --- |
| Quorum | | **0.8** | **0.6** | | **0.4** | | **0.2** | |  |
| **Unit 1** | | 26.1 | 13.6 | | 7.2 | | 2.9 | |  |
| **Unit 2** | | 24.6 | 15.6 | | 9.3 | | 4.2 | |  |
| **Unit 3** | | 15.0 | 9.1 | | 4.9 | | 2 | |  |
| **Unit 4** | | **--** | 7.7 | | 4.7 | | 2 | |  |
| **Unit 5** | | 8.3 | 5.5 | | 3.1 | | 1.3 | |  |
| **Cartilaginous fish** | | |  | |  | |  | |  |
| Quorum | | **0.8** | **0.6** | | **0.4** | | **0.2** | |  |
| **Unit 1** | | 12.2 | 6.7 | | 3.7 | | 1.4 | |  |
| **Unit 2** | | 8.8 | 5.6 | | 3.3 | | 1.5 | |  |
| **Unit 3** | | 3.6 | 2 | | 1.2 | | 0.9 | |  |
| **Unit 4** | | 5.9 | 3.9 | | 2.3 | | 0.8 | |  |
| **Unit 5** | | 5.3 | 3.1 | | 1.8 | | 1 | |  |
| **Bony fish** |  | | |  | |  | |  | |
| Quarum | **0.8** | | | **0.6** | | **0.4** | | **0.2** | |
| **Unit 1** | 9.6 | | | 4.9 | | 2.6 | | 1.2 | |
| **Unit 2** | 7.8 | | | 4.9 | | 2.7 | | 1.2 | |
| **Unit 3** | **--** | | | 3.9 | | 2.3 | | 1 | |
| **Unit 4** | **--** | | | 1.6 | | 1 | | 0.4 | |
| **Unit 5** | 1.5 | | | 1.5 | | 0.5 | | 0.5 | |

| **Marine reptiles** | |  |  |  |
| --- | --- | --- | --- | --- |
| Quarum | **0.8** | **0.6** | **0.4** | **0.2** |
| **Unit 1** | 6.9 | 3.3 | 1.2 | 1.1 |
| **Unit 2** | 6.6 | 3.9 | 2.2 | 0.8 |
| **Unit 3** | **--** | 3.5 | 2 | 0.9 |
| **Unit 4** | **--** | 1.7 | 1.3 | 0.6 |
| **Unit 5** | 1.0 | 1 | 1 | 0.4 |

**3.** **Paleogeography**

The current location of Alabama was placed largely in offshore environments along the coastline of the Appalachian landmass through the Late Cretaceous (Supplementary Fig. S4). Alabama was located near the eastern margin of the Mississippian Embayment during the time. The coastline of Alabama had shifted toward south through the Late Cretaceous due to a series of regression events^9^. During the Late Cretaceous, the Western Interior Seaway started to disappear. Our study area represents the northeastern Gulf of Mexico, which is located about 500 km from the Chicxulub impact site as seen in the main text (Fig. 1).

80.3 Ma (Early Campanian) 73.8 Ma (Late Campanian) 66 Ma (end-Maastrichtian)

**Figure S4.** Paleogeographic maps of North America in Late Cretaceous. **Left.** 80.3 Ma (Early Campanian); **B.** 73.8 Ma (Late Campanian); **C**. 66.0 Ma (Maastrichtian). The position of the paleoshoreline had extended further south in Alabama through the time. Maps modified from Charles Scotese (ref. 10).

**4. Alabama Marine Vertebrates**

A summary of Late Cretaceous marine vertebrate fossil specimens from Alabama can be found in Ikejiri et al. (ref. 4). Semiaquatic and terrestrial taxa, such as crocodilians, pterosaurs, non-avian dinosaurs, and seabirds were not included in this study. All specimens belong to 12 institutions in the U.S. and U.K., and they are listed as the followings: **AMNH**, American Museum of Natural History, New York, NY, USA; **ANSP**, Academy of Natural Sciences of Philadelphia, PA, USA; **ALMNH**, Alabama Museum of Natural History, University of Alabama, Tuscaloosa, AL, USA; **AUMP**, Auburn University Museum of Paleontology, Auburn, AL, USA; **CCK**, Cretaceous research collections at Columbus State University, Columbus, GA, USA; **CMC**, Cincinnati Museum Center, Cincinnati, OH, USA; **FHSM,** Fort Hays State University Sternberg Museum of Natural History, hays, KS, USA; **FMNH**, Field Museum of Natural History, Chicago, IL, USA; **GSA**, Geological Survey of Alabama, Tuscaloosa, AL (vertebrate fossil collection currently housed at UAM) , USA; **LACM**, Natural History Museum of Los Angeles County Museum, Los Angeles, CA, USA; **MMNS**, Mississippi Museum of Natural Science, Jackson, MS, USA; **MSC**, McWane Science Center, Birmingham, AL, USA; **NHMUK**, Natural History Museum in London, United Kingdom; **NJSM**, New Jersey state Museum, Trenton, NJ, USA; **RMM**, Red Mountain Museum, Birmingham, AL (fossil collection currently housed at MSC) , USA; **UWA**, University of West Alabama, Livingston, AL, USA; **UPI**, Museum of Evolution, Uppsala University, Uppsala, Sweden; **USNM**, United States National Museum, Washington D.C., USA; **YPM**, Yale Peabody Museum, New Haven, CT, USA.

| **Table S3.** Taxonomic list of Late Cretaceous marine vertebrates from Alabama with stratigraphic occurrences. Geologic units (formations and members) for the five stratigraphic units (Unit 1 to 5) are given in the main text (Table 1) and Supplementary Figure S1. Abbreviations for higher taxa: **A**: Actinopterygii (ray-finned fish); **C**: Chondrichthyes; **R**: reptiles (Sauropsida); **S**: Sarcopterygii (lobe-finned fish). | | | | | |
| --- | --- | --- | --- | --- | --- |
| **Higher taxa** | **Genus** | **Species** | **Stratigraphic occurrences in AL*** | **Habitats**** | **K–Pg survived in global scale**** |
| Heterodontiformes (C) | *Heterodontus*(?) | sp. | (0)1 | nektonic carnivore | Yes (genus) |
| Hybodontiformes (C) | *Meristodonoides*  (cf. *Hybodus*) | sp. | (0) 2 | nektonic carnivore | No |
| Hybodontiformes (C) | *Lissodus* | sp. | (0)- 2 | nektonic carnivore | No |
| Hybodontiformes (C) | *Lonchidion* | sp. | (0) 2 | nektonic carnivore | No |
| Pachycormiformes (A) | *Belonostomus* | sp. | (0),1 | nektonic carnivore | No |
| Chimaeriformes (C) | *Edaphodon* | *mirificus* | (0),2 | nektobenthic carnivore | Yes (genus) |
| Lamniformes (C) | *Paranomotodon* | *angustidens*(?) | (0),2 | nektonic carnivore | No |
| Lamniformes (C) | *Scapanorhynchus* | *rapax* | (0) 2 | nektonic carnivore | No |
| Myliobatiformes (C) | *Rhombodus* | *binkhorsti* | (0)2 | nektonic carnivore | No (yes for genus) |
| Rajiformes (C) | *Dasyatis* | sp. | (0)-2 | nektonic carnivore | Yes (genus) |
| Sclerorhynchiformes (C) | *Sclerorhynchus* | sp. | (0)-2 | nektonic carnivore | No |
| Aulopiformes (A) | *Cimolichthys* | *nepaholica* | (0)-2 | nektonic carnivore | No |
| Crossognathiformes (A) | *Pachyrhizodus* | *caninus* | (0)-2 | nektonic carnivore | Yes(?) |
| Ichthyodectiformes (A) | *Ichthyodectes* | *ctenodon* | (0)-2 | nektonic carnivore | No |
| Ichthyodectiformes (A) | *Saurocephalus* | sp. | (0)-2 | nektonic carnivore | Yes |
| Mosasauridae (R) | *Prognathodon* | sp. | (0)-2 | aquatic carnivore | No |
| Mosasauridae (R) | *Tylosaurus* | *nepaeolicus*(?) | (0)-2 | aquatic carnivore | No |
| Hybodontiformes (C) | *Ptychodus* | *rugosus* | **1** | nektonic carnivore | No |
| Hybodontiformes (C) | *Ptychodus* | *whipplei* | **1** | nektonic carnivore | No |
| Lamniformes (C) | *Cretodus* | *semplicatus* | **1** | nektonic carnivore | No |
| Orectolobiformes (C) | *Cantioscyllium* | sp. | **1** | nektonic carnivore | No |
| Orectolobiformes (C) | *Chiloscyllium* | *greeni* | **1** | nektonic carnivore | Yes (genus) |
| Rajiformes (C) | *Pseudohypoliphus* | *mcnultyi* | **1** | nektobenthic carnivore | No(?) |
| Rajiformes (C) | *Ptychotrygon* | *triangularis* | **1** | nektobenthic carnivore | No |
| Sclerorhynchiformes (C) | *Borodinopristis* | *schwimmeri* | **1** | nektobenthic carnivore | No |
| Squatiniformes (C) | *Squatina* | *hassei* | **1** | nektobenthic carnivore | No |
| Beryciformes (A) | *Hoplopteryx* | sp. | **2** | nektonic carnivore? | No(?) |
| Tselfatiformes (A) | *Moorevillia* | *hardi* | **2** | nektonic carnivore? | No |
| Tselfatiformes (A) | *Palelops* | *eutawnesis* | **2** | nektonic carnivore? | No |
| Pachycormiformes (A) | *Bonnerichthys* | *gladius* | **2** | nektonic carnivore (or planktivore?) | No |
| Mosasauridae (R) | *Clidastes* | *liodontus*  (cf. *C*. *moorevillensis*) | **2** | aquatic carnivore | No |
| Plesiosauria (R) | polycotylid | species indet. | **2** | aquatic carnivore | No |
| Testudines (R) | *Calcarichelys* | *gemma* | **2** | aquatic omnivore | No |
| Testudines (R) | *Chelosphargis* | *advena* | **2** | aquatic omnivore | No |
| Testudines (R) | *Corsochelys* | *haliniches* | **2** | aquatic omnivore | No |
| Testudines (R) | *Ctenochelys* | *tenuitesta* | **2** | aquatic omnivore | No |
| Testudines (R) | *Lophochelys* | *venatrix* | **2** | aquatic omnivore | No? (the genus survived in the Danian?) |
| Crossognathiformes (A) | *Pachyrhizodus* | *minimus* | 1(?),2 | nektonic carnivore | Yes (genus?) |
| Coelacanthiformes (S) | *Megalocoelacanthus* | *dobiei* | 1(?),2 | nektobenthic carnivore | No |
| Lamniformes (C) | *Scapanorhynchus* | *raphiodon* | 1,2 | nektonic carnivore | No(?) |
| Lamniformes (C) | *Squalicorax* | *falcatus* | 1,2 | nektonic carnivore | No |
| Myliobatiformes (C) | *Brachyrhizodus* | *wichitaensis* | 1,2 | nektobenthic carnivore(?) | No |
| Chimaeriformes (C) | *Edaphodon* | *barberi* | 1,2 | nektobenthic carnivore | Yes (genus) |
| Hybodontiformes (C) | *Ptychodus* | *polygurus* | 1,2 | nektonic carnivore | No |
| Albuliformes (A) | *Albula* | *dunklei* | 1,2 | nektobenthic carnivore | No |
| Pachycormiformes (A) | *Protosphyraena* | *nitida* | 1,2 | nektonic carnivore | No |
| Pycnodontiformes (A) | *Hadrodus* | *priscus* | 1,2 | nektonic durophage-carnivore | No |
| Pycnodontiformes (A) | *Phacodus* | *puncatus* | 1,2 | nektonic durophageo & carnivore | No(?) |
| Tselfatiformes (A) | *Bananogmius* | *crieleyi* | 1,2 | nektonic carnivore | Yes |
| Mosasauridae (R) | *Eonatator* | *sternbergi* | 1,2 | aquatic carnivore | No |
| Mosasauridae (R) | *Platecarpus* | *tympaniticus* | 1,2 | aquatic carnivore | No |
| Mosasauridae (R) | *Selmasaurus* | *russelli* | 1,2 | aquatic carnivore | No |
| Plesiosauria (R) | elasmosaurid | species indet. | 1,2 | aquatic carnivore | No |
| Testudines (R) | *Thinochelys* | *lapisossea* | 1,2 | aquatic omnivore | No |
| Mosasauridae (R) | *Mosasaurus* | *missouriensis*(?) | **3** | aquatic carnivore | No |
| Mosasauridae (R) | *Mosasaurus* | *conodon* | **3** | aquatic carnivore | No |
| Mosasauridae (R) | *Platecarpus* | cf. *somenensis* | **3** | aquatic carnivore | No |
| Hybodontiformes (C) | *Ptychodus* | *mortoni* | 1,2,3 | nektonic carnivore | No |
| Aulopiformes (A) | *Enchodus* | *petrosus* | 1,2,3 | nektonic carnivore | Yes |
| Aulopiformes (A) | *Stratodus* | *apicalis* | 1,2,3 | nektonic carnivore | No |
| Mosasauridae (R) | *Tylosaurus* | *proriger* | 1,2,3 | aquatic carnivore | No |
| Testudines (R) | *Chedighaii* | *baeberi* | 1,2,3 | aquatic omnivore | yes (sister taxon: *Bothremys*) |
| Mosasauridae (R) | *Clidastes* | *propython* | 1(?),2,3 | aquatic carnivore | No |
| Aulopiformes (A) | *Enchodus* | *gladiolus* | 2,3 | nektonic carnivore | Yes |
| Ichthyodectiformes | *Saurodon* | *leanus* | 2,3 | nektonic carnivore | No |
| Mosasauridae (R) | *Globidens* | *alabamaensis* | 2,3 | aquatic carnivore | No |
| Testudines (R) | *Prionochelys* | *matutina* | 2,3 | aquatic omnivore | No |
| Testudines (R) | *Toxochelys* | *moorevillensis* | 2,3 | aquatic omnivore | No |
| Lamniformes (C) | *Pseudocorax* | *laevis* | 1,2,4 | nektonic carnivore | No |
| Ichthyodectiformes (A) | *Xiphactinus* | *audax* | 1,2,4 | nektonic carnivore | No |
| Aulopiformes (A) | *Enchodus* | *ferox* | 2,3,4 | nektonic carnivore | Yes |
| Testudines (R) | *Ctenochelys* | *acris* | 2,4 | aquatic omnivore | No |
| Myliobatiformes (C) | *Pseudohypolophus* | *mcnultyi* | **5** | nektobenthic carnivore | No(?) |
| Orectolobiformes (C) | *Ginglymostoma* | sp. | **5** | nektobenthic carnivore | Yes (genus) |
| Rajiformes (C) | *Sclerorhynchus* | sp. | **5** | nektonic carnivore | No |
| Aulopiformes (A) | *Enchodus* | sp. | 5 | nektonic carnivore | No |
| Lamniformes (C) | *Squalicorax* | *kaupi* | 1,2,3,5 | nektonic carnivore | No |
| Lamniformes (C) | *Cretoxyrhina* | *mantelli* | 1,2,3,5 | nektonic carnivore | No |
| Sclerorhynchiformes? (C) | *Ischyrhiza* | *mira* | 1,2,3,5 | nektonic carnivore | Yes |
| Mosasauridae (R) | *Plioplatecarpus* | sp. | 1,2,3,5 | aquatic carnivore | No |
| Lamniformes (C) | *Cretalamna* | *appendiculata* | 1,2,4,5 | nektonic carnivore | Yes |
| Pycnodontiformes (A) | *Anomoeodus* | *phaseolus* | 1,2,4,5 | nektonic durophage-carnivore | No |
| Lamniformes (C) | *Scapanorhynchus* | *texanus* | 1,2,3,4,5 | nektonic carnivore | No |
| Lamniformes (C) | *Serratolamna* | *serrata* | 1,2,5 | nektonic carnivore | No |
| Lamniformes (C) | *Carcharias*(?) | sp. | 1(?),5 | nektonic carnivore | Yes (genus) |
| Lamniformes (C) | *Squalicorax* | *pristodontus* | 2,3,4,5 | nektonic carnivore | No |
| Testudines (R) | *Protostega* | *gigas* | 2,3,5(?) | aquatic omnivore | No |
| Mosasauridae (R) | *Mosasaurus* | *maximus* | 3,4,5 | aquatic carnivore | No |

*Data updated from Ikejiri et al. (ref. 4). Bold numbers indicate singleton taxa. The occurrence of Unit 0 is based on data from the Western Interior Seaway and other parts of the Gulf of Mexico (see the additional explanation in Methods).

**Data from the Paleobiology Database^11^.

**Table S4.** Marine vertebrate taxa from the Paleocene identified from Alabama. The detail is currently understudied by one of the authors (T.I.).

| **Geologic units** | **Sharks** | **Bony fish** | **Reptile** |
| --- | --- | --- | --- |
| Impact deposits: | *Squalicorax pristodontus*(?); | *Enchodus* sp. | *Mosasaurus maximus* |
| Clayton Fm: | *Cretalamna* sp.; *Sphenodus* sp.; *Striatolamina* sp. | *Enchodus*(?). |  |

**Table S5.** Data on species counts and origination and extinction rates used for Figure 3 and Figure 4 in the main text. Temporarily disappeared taxa (Lazarus taxa) were included. An asterisk mark indicates a significantly high extinction value (based on the upper 95% CI).

**All Vertebrates (with singletons)**

| **Stratigraphic** |  | **species count** |  | **Standing diversity** | **Percentage** | | **Error**  **(lower)** | **bar**  **(upper)** |
| --- | --- | --- | --- | --- | --- | --- | --- | --- |
| **unit** | **Occurred** | **Originate** | **Extinct** |  | **Originate** | **Extinct** | **Extinct** | **Extinct** |
| Santonian (Unit 0) | 12.0 | -- | -- | -- | -- | -- |  |  |
| Unit 1 | 60* | 43* | 9 | 44.0 | 71.67%* | 15.00% | 30.89% | 12.22% |
| Unit 2 | 68* | 20 | 40* | 51.0 | 29.41% | 58.82% | 80.60% | 49.29% |
| Unit 3 | 29 | 4 | 13 | 24.0 | 13.79% | 44.83% | 51.00% | 26.89% |
| Unit 4 | 17 | 0 | 3 | 14.5 | 0.00% | 17.65% | 27.22% | 10.67% |
| Unit 5 | 18 | 4 | 15 | 13.5 | 22.22% | 83.33%* | 96.24% | 61.66% |
| Paleocene | 4 | 3? | 0? | 1.5 | -- | -- |  |  |
| Sum (Unit 1-5) | 192 | 71 | 80 |  |  |  |  |  |
| MEAN | 38.4 | 14.2 | 16 |  | 27.42% | 43.93% |  |  |
| SD | 24.0 | 17.8 | 14.2 |  | 27.05% | 28.74% |  |  |
| 95% CI | 21.0 | 15.6 | 12.4 |  | 23.71% | 25.19% |  |  |
| Upper | 59.4 | 29.8 | 28.4 |  | 51.13% | 69.11% |  |  |
| Lower | 17.4 | -1.4 | 3.6 |  | 3.71% | 18.74% |  |  |

**All Vertebrates (without singletons)**

| **Stratigraphic** |  | | **species count** | |  | | **Standing diversity** | | **Percentage** | | | | **Error**  **(lower)** | | **bar**  **(upper)** | | |
| --- | --- | --- | --- | --- | --- | --- | --- | --- | --- | --- | --- | --- | --- | --- | --- | --- | --- |
| **unit** | **Occurred** | | **Originate** | | **Extinct** | |  | | **Originate** | | **Extinct** | | **Extinct** | | | | **Extinct** |
| Santonian (Unit 0) | | 12 | | -- | | -- | | -- | | -- | | -- | | -- | | -- | |
| Unit 1 | | 45* | | 29* | | 3 | | 17.5 | | 64.44%* | | 6.67% | | 22.23% | | 7.65% | |
| Unit 2 | | 51* | | 9 | | 25* | | 17.5 | | 17.65% | | 49.02% | | 77.23% | | 46.66% | |
| Unit 3 | | 27 | | 1 | | 9 | | 5.0 | | 3.70% | | 33.33% | | 45.17% | | 22.72% | |
| Unit 4 | | 17 | | 0* | | 5 | | 1.5 | | 0.00% | | 29.41% | | 38.10% | | 17.79% | |
| Unit 5 | | 15 | | 0 | | 9 | | 5.5 | | 0.00% | | 60.00%* | | 79.48% | | 48.41% | |
| Paleocene (Unit 6) | | 4? | | 3? | | 0? | | 2 | | -- | | -- | |  | |  | |
| Sum (Unit 1-5) | | 155 | | 39 | | 51 | |  | |  | |  | |  | |  | |
| MEAN | | 31 | | 7.8 | | 10.2 | |  | | 17.16% | | 35.69% | |  | |  | |
| SD | | 16.3 | | 12.4 | | 8.7 | |  | | 27.41% | | 20.35% | |  | |  | |
| 95% CI | | 14.3 | | 10.9 | | 7.6 | |  | | 24.03% | | 17.83% | |  | |  | |
| Upper | | 45.3 | | 18.7 | | 17.8 | |  | | 41.19% | | 53.52% | |  | |  | |
| Lower | | 16.7 | | -3.1 | | 2.6 | |  | | -6.87% | | 17.85% | |  | |  | |

Table S5. (*cont*.)

**Cartilaginous fishes (with singletons)**

| **Stratigraphic** | **Species count (#s)** | | |  | **Percentage** | | **Error** | **bar** |
| --- | --- | --- | --- | --- | --- | --- | --- | --- |
| **Unit** |  |  |  |  |  |  | **(lower)** | **(upper)** |
|  | **Occurred** | **Originate** | **Extinct** |  | **Origination** | **Extinction** | **Extinction** | **Extinction** |
| Unit 1 | 33* | 23* | 11 |  | 69.70%* | 33.33% | 46.34% | 23.55% |
| Unit 2 | 22 | 1 | 13* |  | 4.55% | 59.09% | 76.11% | 45.79% |
| Unit 3 | 10 | 0 | 1 |  | 0.00% | 10.00% | 30.88% | 12.22% |
| Unit 4 | 8 | 0 | 1 |  | 0.00% | 12.50% | 20.96% | 6.92% |
| Unit 5 | 10 | 3 | 9 |  | 30.00% | 90.00%* | 110.63% | 72.37% |
| Sum | 83 | 27 | 35 |  |  |  |  |  |
| MEAN | 16.6 | 5.4 | 7 |  | 20.85% | 40.98% |  |  |
| SD | 10.7 | 9.9 | 5.7 |  | 30.02% | 33.77% |  |  |
| 95% CI | 9.4 | 8.7 | 5.0 |  | 26.31% | 29.60% |  |  |
| Upper | 26.0 | 14.1 | 12.0 |  | 47.16% | 70.58% |  |  |
| Lower | 7.2 | -3.3 | 2.0 |  | -5.47% | 11.39% |  |  |

**Cartilaginous fishes (without singletons)**

| **Stratigraphic** | **Species count (#s)** | | |  | **Percentage** | | **Error** | **bar** |
| --- | --- | --- | --- | --- | --- | --- | --- | --- |
| **Unit** |  |  |  |  |  |  | **(lower)** | **(upper)** |
|  | **Occurred** | **Originate** | **Extinct** |  | **Origination** | **Extinction** | **Extinction** | **Extinction** |
| Unit 1 | 20* | 8* | 1 |  | 40.00%* | 5.00% | 29.67% | 12.22% |
| Unit 2 | 20* | 1 | 10* |  | 5.00% | 50.00% | 90.67% | 57.22% |
| Unit 3 | 10 | 0 | 1 |  | 0.00% | 10.00% | 4.80% | 17.08% |
| Unit 4 | 8 | 0 | 1 |  | 0.00% | 12.50% | 18.39% | 4.80% |
| Unit 5 | 8 | 0 | 5 |  | 0.00% | 62.50%* | 83.97% | 51.92% |
| Sum | 66 | 9 | 18 |  |  |  |  |  |
| MEAN | 13.2 | 1.8 | 3.6 |  | 9.00% | 28.00% |  |  |
| SD | 6.3 | 3.5 | 4.0 |  | 17.46% | 26.30% |  |  |
| 95% CI | 5.5 | 3.1 | 3.5 |  | 15.31% | 23.06% |  |  |
| Upper | 18.7 | 4.9 | 7.1 |  | 24.31% | 51.06% |  |  |
| Lower | 7.7 | -1.3 | 0.1 |  | -6.31% | 4.94% |  |  |

Table S5. (*cont*.)

**Bony fishes (with singletons)**

| **Stratigraphic** | **Species count (#s)** | | |  | **Percentage** | | **Error** | **bar** |
| --- | --- | --- | --- | --- | --- | --- | --- | --- |
| **Unit** |  |  |  |  |  |  | **(lower)** | **(upper)** |
|  | **Occurred** | **Originate** | **Extinct** |  | **Origination** | **Extinction** | **Extinction** | **Extinction** |
| Unit 1 | 17 | 11* | 1 |  | 64.71%* | 5.88% | 11.67% | 2.20% |
| Unit 2 | 22* | 6 | 15* |  | 27.27% | 68.18%* | 86.21% | 53.69% |
| Unit 3 | 7 | 0 | 2 |  | 0.00% | 28.57% | 23.49% | 8.40% |
| Unit 4 | 5 | 0 | 1 |  | 0.00% | 20.00% | 30.88% | 12.22% |
| Unit 5 | 5 | 1 | 2 |  | 20.00% | 40.00% | 54.47% | 28.58% |
| Sum | 56 | 18 | 21 |  |  |  |  |  |
| MEAN | 11.2 | 3.6 | 4.2 |  | 22.40% | 32.53% |  |  |
| SD | 7.8 | 4.8 | 6.1 |  | 26.57% | 23.50% |  |  |
| 95% CI | 6.9 | 4.2 | 5.3 |  | 23.28% | 20.60% |  |  |
| Upper | 18.1 | 7.8 | 9.5 |  | 45.68% | 53.13% |  |  |
| Lower | 4.3 | -0.6 | -1.1 |  | -0.89% | 11.93% |  |  |

**Bony fishes (without singletons)**

| **Stratigraphic** | **Species count (#s)** | | |  | **Percentage** | | **Error** | **bar** |
| --- | --- | --- | --- | --- | --- | --- | --- | --- |
| **Unit** |  |  |  |  |  |  | **(lower)** | **(upper)** |
|  | **Occurred** | **Originate** | **Extinct** |  | **Origination** | **Extinction** | **Extinction** | **Extinction** |
| Unit 1 | 15* | 12* | 1 |  | 80.00%* | 6.67% | 17.08% | 4.80% |
| Unit 2 | 17* | 3 | 10* |  | 17.65% | 58.82%* | 77.23% | 46.66% |
| Unit 3 | 7 | 0 | 3 |  | 0.00% | 42.86% | 40.47% | 19.42% |
| Unit 4 | 5 | 0 | 2 |  | 0.00% | 40.00% | 54.47% | 28.58% |
| Unit 5 | 4 | 0 | 1 |  | 0.00% | 25.00% | 36.90% | 16.18% |
| Sum | 48 | 15 | 17 |  |  |  |  |  |
| MEAN | 9.6 | 3 | 3.4 |  | 19.53% | 34.67% |  |  |
| SD | 6.0 | 5.2 | 3.8 |  | 34.66% | 19.73% |  |  |
| 95% CI | 5.2 | 4.6 | 3.3 |  | 30.38% | 17.29% |  |  |
| Upper | 14.8 | 7.6 | 6.7 |  | 49.91% | 51.96% |  |  |
| Lower | 4.4 | -1.6 | 0.1 |  | -10.85% | 17.38% |  |  |

Table S5. (*cont*.)

**Marine reptiles (with singletons)**

| **Stratigraphic** | **Species count (#s)** | | |  | **Percentage** | | **Error** | **bar** |
| --- | --- | --- | --- | --- | --- | --- | --- | --- |
| **Unit** |  |  |  |  |  |  | **(lower)** | **(upper)** |
|  | **Occurred** | **Originate** | **Extinct** |  | **Origination** | **Extinction** | **Extinction** | **Extinction** |
| Unit 1 | 10 | 9 | 0 |  | 90.00%* | 0.00% | 3.69% | 0.00% |
| Unit 2 | 24* | 13* | 15* |  | 54.17% | 62.50% | 79.48% | 48.41% |
| Unit 3 | 13 | 4 | 9 |  | 30.77% | 69.23% | 87.32% | 54.57% |
| Unit 4 | 4 | 0 | 1 |  | 0.00% | 25.00% | 36.90% | 16.18% |
| Unit 5 | 3 | 0 | 3 |  | 0.00% | 100.00%* | 121.63% | 81.36% |
| Sum | 54 | 26 | 28 |  |  |  |  |  |
| MEAN | 10.8 | 5.2 | 5.6 |  | 34.99% | 51.35% |  |  |
| SD | 8.5 | 5.7 | 6.3 |  | 38.28% | 39.19% |  |  |
| 95% CI | 7.4 | 5.0 | 5.5 |  | 33.55% | 34.35% |  |  |
| Upper | 18.2 | 10.2 | 11.1 |  | 68.54% | 85.69% |  |  |
| Lower | 3.4 | 0.2 | 0.1 |  | 1.44% | 17.00% |  |  |

**Marine reptiles (without singletons)**

| **Stratigraphic** | **Species count (#s)** | | |  | **Percentage** | | **Error bar** | **Error bar** |
| --- | --- | --- | --- | --- | --- | --- | --- | --- |
| **Unit** |  |  |  |  |  |  | **lower** | **upper** |
|  | **Occurred** | **Originate** | **Extinct** |  | **Origination** | **Extinction** | **extinction** | **extinction** |
| Unit 1 | 10 | 9* | 1 |  | 90.00%* | 10.00% | 14.42% | 3.45% |
| Unit 2 | 14* | 5 | 5 |  | 35.71% | 35.71% | 55.62% | 30.27% |
| Unit 3 | 10 | 1 | 6* |  | 10.00% | 60.00% | 77.23% | 45.79% |
| Unit 4 | 4 | 0 | 2 |  | 0.00% | 50.00% | 65.92% | 37.11% |
| Unit 5 | 3 | 0 | 3 |  | 0.00% | 100.00%* | 121.63% | 81.36% |
| Sum | 41 | 15 | 17 |  |  |  |  |  |
| MEAN | 8.2 | 3 | 3.4 |  | 27.14% | 51.14% |  |  |
| SD | 4.6 | 3.9 | 2.1 |  | 38.05% | 33.16% |  |  |
| 95% CI | 4.0 | 3.5 | 1.8 |  | 33.35% | 29.06% |  |  |
| Upper | 12.2 | 6.5 | 5.2 |  | 60.50% | 80.21% |  |  |
| Lower | 4.2 | -0.5 | 1.6 |  | -6.21% | 22.08% |  |  |

**Table S6.** Extinction rates of all marine vertebrates based on the data excluding singleton taxa. The data of *p* and *q* are used in Figure 5.

**A. All vertebrates**

| **Quantity** | **symbol ^(1)^** | **Unit 1** | **Unit 2** | **Unit 3** | **Unit 4** | **Unit 5** |
| --- | --- | --- | --- | --- | --- | --- |
| **Duration in million years (m.y.)** | k | 3 m.y. | 5 m.y. | 8 m.y. | 2 m.y. | 4 m.y. |
| **Singleton taxa** |  | 15 | 17 | 3 | 0 | 3 |
| **# of taxa crossing both lower and upper boundaries** | N bt | 9 | 18 | 16 | 14 | 4 |
| **Originate # (w/o singletons)** | N b | 6 | 9 | 1 | 0 | 0 |
| **Extinct # (w/o singletons)** | N t | 6 | 26 | 9 | 3 | 11 |
| **Standing diversity (w/o singletons)** |  | 6.0 | 17.5 | 5.0 | 1.5 | 5.5 |
| **Proportional origination** | PO | 0.67 | 0.50 | 0.06 | 0.00 | 0.00 |
| **Proportional extinction** | PE | 0.67 | 1.44 | 0.56 | 0.21 | 2.75 |
| **Proportional origination rate per-m.y.** | PO m.y. | 0.22 | 0.10 | 0.01 | 0.00 | 0.00 |
| **Proportional extinction rate per-m.y.** | PE m.y. | 0.22 | 0.29 | 0.07 | 0.11 | 0.69 |
| **Per-capita origination rate (per Lmy)** | *p* | -0.14 | -0.14 | -0.35 | -- | -- |
| **Per-capita extinction rate (per Lmy)** | *q* | -0.14 | 0.07 | -0.07 | -0.77 | 0.25 |

^(1)^ Equivalences of symbols are from refs. 12 and 13.

**B. Cartilaginous fish**

| **Unit** | **symbol ^(1)^** | **Unit 1** | **Unit 2** | **Unit 3** | **Unit 4** | **Unit 5** |
| --- | --- | --- | --- | --- | --- | --- |
| **Singleton taxa** |  | 13 | 2 | 0 | 0 | 2 |
| **# of taxa crossing both lower and upper boundaries** | N bt | 4 | 9 | 8 | 7 | 1 |
| **Originate # (w/o singletons)** | N b | 1 | 10 | 1 | 1 | 5 |
| **Extinct # (w/o singletons)** | N t | 1 | 10 | 1 | 1 | 5 |
| **Standing diversity (w/o singletons)** | N st | 4.5 | 5.5 | 0.5 | 0.5 | 2.5 |
| **Proportional origination** | PO | 2.00 | 0.11 | 0.00 | 0.00 | 0.00 |
| **Proportional extinction** | PE | 0.25 | 1.11 | 0.13 | 0.14 | 5.00 |
| **Proportional origination rate per-m.y.** | PO m.y. | 0.67 | 0.02 | 0.00 | 0.00 | 0.00 |
| **Proportional extinction rate per-m.y.** | PE m.y. | 0.08 | 0.22 | 0.02 | 0.07 | 1.25 |
| **Per-capita origination rate (per Lmy)** | *p* | 0.23 | -0.44 | -- | -- | -- |
| **Per-capita extinction rate (per Lmy)** | *q* | -0.46 | 0.02 | -0.26 | -0.97 | 0.40 |

^(1)^ Equivalences of symbols are from refs. 12 and 13.

**Table S6 (cont.)**

**C. Bony fish**

| **Unit** | **symbol ^(1)^** | **Unit 1** | **Unit 2** | **Unit 3** | **Unit 4** | **Unit 5** |
| --- | --- | --- | --- | --- | --- | --- |
| **Singleton taxa** |  | 5 | 0 | 0 | 1 | 0 |
| **# of taxa crossing both lower and upper boundaries** | N bt | 14 | 7 | 4 | 3 | 3 |
| **Originate #** **(w/o singletons)** | N b | 12 | 3 | 0 | 0 | 0 |
| **Extinct #** **(w/o singletons)** | N t | 1 | 10 | 3 | 2 | 1 |
| **Standing diversity (w/o singletons)** | N st | 6.5 | 6.5 | 1.5 | 1 | 0.5 |
| **Proportional origination** | PO | 0.86 | 0.43 | 0.00 | 0.00 | 0.00 |
| **Proportional extinction** | PE | 0.07 | 1.43 | 0.75 | 0.67 | 0.33 |
| **Proportional origination rate per-m.y.** | PO m.y. | 0.29 | 0.09 | 0.00 | 0.00 | 0.00 |
| **Proportional extinction rate per-m.y.** | PE m.y. | 0.02 | 0.29 | 0.09 | 0.33 | 0.08 |
| **Per-capita origination rate (per Lmy)** | *p* | -0.05 | -0.17 | -- | -- | -- |
| **Per-capita extinction rate (per Lmy)** | *q* | -0.88 | 0.07 | -0.04 | -0.20 | -0.27 |

^(1)^ Equivalences of symbols are from refs. 12 and 13.

**D. Marine reptiles**

| **Unit** | **symbol ^(1)^** | **Unit 1** | **Unit 2** | **Unit 3** | **Unit 4** | **Unit 5** |
| --- | --- | --- | --- | --- | --- | --- |
| **Singleton taxa** |  | 0 | 10 | 3 | 0 | 0 |
| **# of taxa crossing both lower and upper boundaries** | N bt | 1 | 3 | 3 | 3 | 0 |
| **Originate # (w/o singletons)** | N b | 9 | 5 | 1 | 0 | 0 |
| **Extinct # (w/o singletons)** | N t | 1 | 5 | 6 | 2 | 3 |
| **Standing diversity (w/o singletons)** | N st | 5 | 5 | 3.5 | 1 | 1.5 |
| **Proportional origination** | PO | 9.00 | 1.67 | 0.33 | 0.00 | -- |
| **Proportional extinction** | PE | 1.00 | 1.67 | 2.00 | 0.67 | -- |
| **Proportional origination rate per-m.y.** | PO m.y. | 3.00 | 0.33 | 0.04 | 0.00 | -- |
| **Proportional extinction rate per-m.y.** | PE m.y. | 0.33 | 0.33 | 0.25 | 0.33 | -- |
| **Per-capita origination rate (per Lmy)** | *p* | 0.73 | 0.10 | -0.14 | -- | -- |
| **Per-capita extinction rate (per Lmy)** | *q* | 0.00 | 0.10 | 0.09 | -0.20 | -- |

^(1)^ Equivalences of symbols are from refs. 12 and 13.

**5. Global Extinction Pattern of Cretaceous Marine Vertebrates**

On a worldwide scale, a total of 396 genera of marine vertebrates were recorded from the Cenomanian to Paleocene. Of the 690 total occurrences, the total generic counts show no significant decline (95% CI) through the Maastrichtian–Paleocene boundary (Table S7 and Table S8). Of the three vertebrate groups, a significant-level of the decline occurred only in reptiles from the Maastrichtian to the Paleocene, indicating that marine reptiles (esp., mosasaurs and plesiosaurs) faced severe damage. To some degree, this marine vertebrate extinction pattern on the global scale resembles the pattern found in Alabama fauna.

**Table S7.** The generic-level occurrence of Late Cretaceous and Paleocene marine vertebrates on a global scale. Data were downloaded from the Paleobiology Database^11^ (accessed in January 2019). Questionable and possible non-marine taxa were not included. Vertebrate genera used for this analysis are listed in Supplementary Table S8.

|  | **All vertebrates** | **Cartilaginous fishes** | **Bony fishes** | **Reptiles** |
| --- | --- | --- | --- | --- |
| **Cenomanian** | 108* | 44* | 39 | 25 |
| **Santonian** | 133 | 47 | 33 | 53 |
| **Campanian** | 151 | 82 | 31 | 38 |
| **Maastrichtian** | 154 | 69 | 29 | 56 |
| **Paleocene** | 144 | 64 | 58 | 22* |
| **Total occurrence** | 690 | 306 | 190 | 194 |
| **Mean** ± **SD**  **95% CI (lower limit)** | 138.0±18.6  119.4 | 61.2±15.8  45.4 | 38.0±11.8  26.2 | 38.8±15.6  23.2 |
| **Total genera count** | 396 | 139 | 124 | 133 |
| **K–Pg victims** | 102 | 39 | 17 | 46 |
| **K–Pg survivors** | 51 | 31 | 11 | 9 |
| **Newly appeared genera in Paleocene** | 93 | 33 | 47 | 13 |

*Asterisk symbols indicate significantly low counts based on the 95% CI.

**Table S8.** A list of marine vertebrate genera from the Late Cretaceous to Paleocene on a global scale. Data are selected from the Paleobiology Database (ref. 11; <http://fossilworks.org>). A summary of generic counts through the K–Pg boundary can be found in Table S5. Total numbers of species are also listed for each genus from the database, but alpha taxonomy needs to be clarified for some of them. As such, specific names are not listed (available in the database) and analyzed in this study. **Abbreviations for ages: Ce**: Cenomanian; **Sa**: Santonian; **Ca**: Campanian; **Ma**: Maastrichtian; **Pa**: Paleocene. Taxa with an asterisk mark indicate the pre-Unit 1 (i.e., Unit 0: early to mid-Santonian) occurrence, which is used for Lazarus taxon counts (see ‘0’ occurrence in Supplementary Table S3).

**(a). Cartilaginous fishes**

| **Chondrichthyes** | **Genus** | **#s of species** | **Age** |
| --- | --- | --- | --- |
| Chimaeriformes | *Edaphodon** | 7 | Ce–Pa |
| Chimaeriformes | *Ischyodus** | 4 | Ce–Pa |
| Chimaeriformes | *Elasmodus* | 1 | Ce–Ma |
| Heterodontiformes | *Heterodontus** | 4 | Ce–Pa |
| Hexanchiformes | *Heptranchias* | 1 | Pa |
| Hexanchiformes | *Hexanchus* | 4 | Sa–Pa |
| Hexanchiformes | *Notidanodon* | 3 | Ca–Pa |
| Hexanchiformes | *Notorhynchus* | 1 | Pa |
| Hexanchiformes | *Weltonia* | 1 | Pa |
| Hexanchiformes | *Chlamydoselachus* | 1 | Pa |
| Hybodontiformes | *Asteracanthus* | 1 | Ca |
| Hybodontiformes | *Hybodus** | 4 | Ce–Ma |
| Hybodontiformes | *Lissodus(* | 2 | Sa–Ma |
| Hybodontiformes | *Lonchidion* | 1 | Ma |
| Hybodontiformes | *Polyacrodus* | 1 | Ce–Ca |
| Hybodontiformes | *Ptychodus** | 8 | Ce–Ca |
| Carchariniformes | *Physogaleus* | 1 | Pa |
| Carchariniformes | *Crassescyliorhinus* | 1 | Ca |
| Carchariniformes | *Foumtizia* | 1 | Pa |
| Carchariniformes | *Premontreia* | 1 | Pa |
| Carchariniformes | *Pteroscyllium* | 2 | Ca–Ma |
| Carchariniformes | *Abdounia* | 3 | Pa |
| Carchariniformes | *Galeorhinus* | 5 | Ca–Pa |
| Carchariniformes | *Pachygaleus* | 1 | Pa |
| Carchariniformes | *Palaeogaleus* | 5 | Ca–Pa |
| Carchariniformes | *Paratriakis* | 1 | Sa–Ca |
| Carchariniformes | *Protoscyliorhinus* | 1 | Ce |
| Carchariniformes | *Scyliorhinus* | 8 | Ca–Pa |
| Carchariniformes | *Squatigaleus* | 1 | Ca–Ma |
| Carchariniformes | *Triakis* | 1 | Pa |
| Lamniformes | *Pseudocorax* | 3 | Sa–Ma |
| Lamniformes | *Squalicorax* | 11 | Ce–Pa(?) |
| Lamniformes | *Archaeolamna* | 2 | Ce–Ca |
| Lamniformes | *Cardabiodon* | 1 | Ce |
| Lamniformes | *Cretalamna* | 8 | Ca |
| Lamniformes | *Cretodus* | 4 | Ce–Ma |
| Lamniformes | *Cretoxyrhina* | 1 | Ce–Ca |
| Lamniformes | *Dallasiella* | 1 | Ce |
| Lamniformes | *Plicatolamna* | 2 | Ce–Ma |
| Lamniformes | *Serratolamna* | 3 | Ca–Ma |
| Lamniformes | *Leptostyrax* | 1 | Sa |
| Lamniformes | *Protolamna* | 3 | Ce–Ca |
| Lamniformes | *Oxyrhina* | 4 | Ca–Pa |
| Lamniformes | *Hypotodus* | 2 | Ca–Ma |
| Lamniformes | *Jaekelotodus* | 1 | Pa |
| Lamniformes | *Palaeohypotodus* | 2 | Ma–Pa |
| Lamniformes | *Carcharodon* | 2 | Pa |
| Lamniformes | *Corax* | 1 | Ca |
| Lamniformes | *Isurus* | 2 | Ce–Pa |
| Lamniformes | *Lamna* | 7 | Ce–Pa |
| Lamniformes | *Orthacodus* | 1 | Pa |
| Lamniformes | *Anomotodon* | 3 | Sa–Ma |
| Lamniformes | *Woellsteinia* | 1 | Pa |
| Lamniformes | *Brachycarcharias* | 1 | Pa |
| Lamniformes | *Carcharias* | 16 | Ce–Pa |
| Lamniformes | *Cenocarcharias* | 2 | Ce |
| Lamniformes | *Eostriatolamia* | 1 | Ce–Ca |
| Lamniformes | *Odontaspis* | 17 | Ce–Pa |
| Lamniformes | *Pseudodontaspis* | 3 | Ca–Pa |
| Lamniformes | *Pseudoisurus* | 2 | Ce |
| Lamniformes | *Striatolamia* | 3 | Pa |
| Lamniformes | *Synodontaspis* | 3 | Sa–Ma |
| Lamniformes | *Cretalamna* | 2 | Ce–Ca |
| Lamniformes | *Palaeocarcharodon* | 1 | Pa |
| Lamniformes | *Scapanorhynchus** | 6 | Ce–Pa(?) |
| Lamniformes | *Paranomotodon** | 1 | Sa–Ma |
| [Selachii](https://paleobiodb.org/cgi-bin/bridge.pl?a=checkTaxonInfo&taxon_no=218949&is_real_user=1) | *Mustelus* | 1 | Pa |
| Selachii | *Sphyrna* | 1 | Pa |
| Myliobatiformes | *Coupatezia* | 1 | Pa |
| Myliobatiformes | *Dasyatis** | 10 | Ma–Pa |
| Myliobatiformes | *Igdabatis* | 2 | Ma |
| Myliobatiformes | *Myliobatis* | 1 | Pa |
| Myliobatiformes | *Rhinoptera* | 1 | Ca–Pa |
| Myliobatiformes | *Rhombodus* | 1 | Ca |
| Myliobatiformes | *Hypolophites* | 1 | Pa |
| Myliobatiformes | *Hypolophodon* | 1 | Pa |
| Myliobatiformes | *Palaeodasyatis* | 1 | Pa |
| Myliobatiformes | *Viperecucullus* | 1 | Pa |
| Myliobatiformes | *Aetobatus* | 2 | Pa |
| Myliobatiformes | *Igdabatis* | 2 | Ma |
| Myliobatiformes | *Myliobatis* | 7 | Ma–Pa |
| Myliobatiformes | *Pseudohypolophus* | 2 | Sa–Ca |
| Myliobatiformes | *Pucabatis* | 1 | Ma |
| Myliobatiformes | *Rhinoptera* | 1 | Pa |
| Myliobatiformes | *Rhombodus** | 6 | Ca–Pa |
| Myliobatiformes | *Brachyrhizodus* | 2 | Sa–Ma |
| Myliobatiformes | *Coupatezia* | 2 | Ca–Pa |
| Myliobatiformes | *Texabatis* | 1 | Ma |
| Myliobatiformes | *Turoniabatis* | 1 | Ce |
| Pristiformes | *Onchopristis* | 1 | Ce |
| Pristiformes | *Peyeria* | 1 | Ce |
| Pristiformes | *Pristis* | 1 | Pa |
| Orectolobiformes | *Ginglymostoma* | 3 | Pa |
| Pristiformes | *Cyclobatis* | 3 | Ce |
| Pristiformes | *Cederstroemia* | 1 | Ca |
| Pristiformes | *Dalpiazia* | 1 | Ca–Ma |
| Pristiformes | *Onchopristis* | 2 | Ce–Ca |
| Pristiformes | *Pristis* | 1 | Pa |
| Pristiformes - Orectolobiformes | *Ginglymostoma* | 2 | Ca–Ma |
| Pristiformes - Orectolobiformes | *Cantioscyllium* | 3 | Ce–Ma |
| Pristiformes - Orectolobiformes | *Nebrius* | 1 | Ma–Pa |
| Pristiformes - Orectolobiformes | *Plicatoscyllium* | 4 | Ca–Ma |
| Pristiformes - Orectolobiformes | *Chiloscyllium* | 4 | Sa–Ma |
| Pristiformes - Orectolobiformes | *Hemiscyllium* | 1 | Ca–Ma |
| Pristiformes - Orectolobiformes | *Almascyllium* | 1 | Ce–Sa |
| Pristiophoriformes | *Pristiophorus* | 2 | Sa–Ma |
| Squaliformes | *Centrophoroides* | 2 | Ca |
| Squaliformes | *Centrophorus* | 1 | Ca–Pa |
| Squaliformes | *Centroscymnus* | 4 | Ca–Ma |
| Squaliformes | *Dalatias* | 1 | Pa |
| Squaliformes | *Eoetmopterus* | 1 | Ca |
| Squaliformes | *Megasqualus* | 1 | Pa |
| Squaliformes | *Procentrophorus* | 1 | Ce |
| Squaliformes | *Protosqualus* | 1 | Ce–Ma |
| Squaliformes | *Pseudoechinorhinus* | 2 | Pa |
| Squaliformes | *Squaliodalatias* | 1 | Ce |
| Squaliformes | *Squalus* | 4 | Ce–Pa |
| Synechodontiformes | *Paraorthacodus* | 3 | Ce–Pa |
| Synechodontiformes | *Synechodus* | 8 | Ce–Pa |
| Rajiformes | *Hypolophus* | 1 | Ce–Ca |
| Rajiformes | *Ischyrhiza* | 10 | Sa–Pa |
| Rajiformes | *Tethybatis* | 1 | Ca–Ma |
| Rajiformes | *Raja* | 3 | Ce–Ma |
| Rajiformes | *Erguitaia* | 2 | Ca–Ma |
| Rajiformes | *Hamrabatis* | 2 | Ca–Ma |
| Rajiformes | *Parapalaeobates* | 2 | Ca–Ma |
| Rajiformes | *Paratrygonorrhina* | 1 | Ca |
| Rajiformes | *Protoplatyrhina* | 1 | Ca–Ma |
| Rajiformes | *Rhinobatos* | 1 | Ca–Pa |
| Rajiformes | *Rhinobatos* | 14 | Ce–Pa |
| Rajiformes | *Rhombopterygia* | 1 | Ce |
| Sclerorhynchiformes | *Ankistrorhynchus* | 2 | Sa–Ca |
| Sclerorhynchiformes | *Borodinopristis* | 1 | Sa–Ca |
| Sclerorhynchiformes | *Ctenopristis* | 2 | Ca–Pa |
| Sclerorhynchiformes | *Ganopristis* | 1 | Ca–Ma |
| Sclerorhynchiformes | *Micropristis* | 1 | Ce |
| Sclerorhynchiformes | *Ptychotrygon* | 10 | Ce–Ma |
| Sclerorhynchiformes | *Schizorhiza* | 1 | Ca–Ma |
| Sclerorhynchiformes(?) | *Sclerorhynchus** | 3 | Sa–Ma |

**(b). Bony fishes**

| **Actinopterygii** | **Genus** | **#s of species** | **Age** |
| --- | --- | --- | --- |
| Acipenseriformes | *Acipenser* | 3 | Ca–Pa |
| Acipenseriformes | *Propenser* | 1 | Sa–Pa |
| Albuliformes | *Albula* | 3 | Sa–Pa |
| Albuliformes | *Albulidarum* | 1 | Pa |
| Albuliformes | *Anogmius* | 1 | Sa–Ca |
| Albuliformes | *Cretalbula* | 1 | Ce |
| Albuliformes | *Farinichthys* | 1 | Pa |
| Albuliformes | *Lebonichthys* | 1 | Ce |
| Albuliformes | *Moorevillia* | 1 | Sa |
| Albuliformes | *Pterothrissus* | 1 | Pa |
| Albuliformes | *Pteralbula* | 1 | Pa |
| Araripichthyidae | *Araripichthys* | 1 | Sa |
| Alepisauriformes | *Apateodus* | 1 | Ce–Ma |
| Argentiniformes | *Argentina* | 2 | Pa |
| Argentiniformes | *Protoargentinolithus* | 1 | Pa |
| Argentiniformes | *Protoargentinolithus* | 1 | Pa |
| Anguilliformes | *Rhynchoconger* | 1 | Pa |
| Anguilliformes | *Rhechias* | 1 | Pa |
| Anguilliformes | *Conger* | 1 | Pa |
| Anguilliformes | *Urenchelys* | 1 | Sa |
| Anguilliformes | *Luenchelys* | 1 | Ce |
| Anguilliformes | *Pseudoegertonia* | 1 | Ca–Pa |
| Aulopiformes | *Chlorophthalmus* | 1 | Pa |
| Aulopiformes? | *Stratodus* | 1 | Ce–Ma |
| Aulipiformes | *Cimolichthys* | 1 | Sa–Ca |
| Aulipiformes | *Enchodus* | 17 | Ce–Pa |
| Aulopiformes? | *Eurypholis* | 1 | Ce |
| Aulopiformes? | *Parenchodus* | 1 | Ce |
| Aulopiformes | *Serrilepis* | 3 | Ce |
| Beryciformes | *Beryx* | 1 | Ca–Ma |
| Beryciformes | *Centroberyx* | 3 | Pa |
| Beryciformes | *Hoplostethus* | 1 | Pa |
| Beryciformes | *Judeoberyx* | 1 | Ce |
| Beryciformes | *Paracentrus* | 1 | Ce |
| Beryciformes | *Hoplopteryx* | 1 | Sa–Ca |
| Beryciformes | *Trachichthyidarum* | 1 | Pa |
| Carangiformes | *Carangidarum* | 1 | Pa |
| Clupeiformes | *Clupeidarum* | 1 | Pa |
| Clupeiformes | *Armigatus* | 2 | Ce |
| Crossognathiformes | *Apsopelix* | 1 | Sa |
| Dercetidae | *Cylindracanthus* | 1 | Ca–Pa |
| Dercetidae | *Dercetis* | 1 | Ma |
| Dercetidae | *Rhynchodercetis* | 3 | Ce |
| Ellimmichthyiformes | *Rhombichthys* | 1 | Ce |
| Ellimmichthyiformes | *Triplomystus* | 2 | Ce |
| Ellimmichthyiformes | *Tycheroichthys* | 1 | Ce |
| Elopiformes | *Ctenodentelops* | 1 | Ce |
| Elopiformes | *Elopopsis* | 1 | Ce |
| Elopiformes | *Palelops* | 1 | Sa–Ca |
| Elopiformes | *Pachyrhizodus** | 4 | Ce–Pa(?) |
| Elopiformes | *Paralbula* | 1 | Ce–Ma |
| Elopiformes | *Egertonia* | 1 | Ca |
| Elopiformes | *Osmeroides* | 1 | Sa |
| Esociformes | *Estesesox* | 1 | Ca |
| Gadiformes | *Protocolliolus* | 1 | Pa |
| Gadiformes | *Gadomorpholithus* | 1 | Pa |
| Gadiformes | *Molva* | 1 | Pa |
| Gadiformes | *Coryphaenoides* | 1 | Pa |
| Gadiformes | *Hymenocephalus* | 1 | Pa |
| Gadiformes | *Palaeogadus* | 1 | Pa |
| Gadiformes | *Raniceps* | 1 | Pa |
| Gadiformes | *Maorigadus* | 1 | Pa |
| Gonorhynchiformes | *Judeichthys* | 1 | Ce |
| Gonorhynchiformes | *Ramallichthys* | 1 | Ce |
| Ichthyodectiformes | *Ghrisichthys* | 1 | Sa |
| Ichthyodectiformes | *Ichthyodectes** | 1 | Ce -Ca |
| Ichthyodectiformes | *Xiphactinus** | 2 | Ce–Ma |
| Ichthyodectiformes | *Gillicus* | 1 | Sa–Ca |
| Ichthyodectiformes | *Saurocephalus* | 1 | Ce–Ma |
| Ichthyodectiformes | *Saurodon** | 2 | Sa–Ma |
| Istiophoriformes | *Xiphias* | 1 | Pa |
| Kurtiformes | *Apogonidarum* | 1 | Pa |
| Labriformes | *Phyllodus* | 1 | Pa |
| Lepisosteiformes | *Atractosteus* | 1 | Ca |
| Ophidiiformes | *Bidenichthys* | 1 | Pa |
| Ophidiiformes | *Dinematichthys* | 1 | Pa |
| Ophidiiformes | *Ogilbia* | 1 | Pa |
| Ophidiiformes | *Fierasferoides* | 1 | Pa |
| Ophidiiformes | *Onuxodon* | 1 | Pa |
| Ophidiiformes | *Ampheristus* | 1 | Pa |
| Ophidiiformes | *Gadophycis* | 1 | Pa |
| Ophidiiformes | *Hoplobrotula* | 1 | Pa |
| Ophidiiformes | *Preophidion* | 1 | Pa |
| Osteoglossiformes | *Brychaetus* | 1 | Ma–Pa |
| Osteoglossiformes | *Genartina* | 1 | Pa |
| Pachycormiformes | *Belonostomus** | 1 | Ma |
| Pachycormiformes | *Protosphyraena* | 2 | Ce–Ma |
| Perciformes | *Scorpaena* | 1 | Pa |
| Perciformes | *Palaeopercichthys* | 1 | Pa |
| Pycnodontiformes | *Hensodon* | 1 | Ce |
| Pycnodontiformes | *Palaeobalistum* | 1 | Ma |
| Pycnodontiformes | *Nursallia* | 1 | Ce |
| Pycnodontiformes | *Palaeobalistum* | 1 | Ce |
| Pycnodontiformes | *Akromystax* | 1 | Ce |
| Pycnodontiformes | *Anomoeodus* | 3 | Sa–Ma |
| Pycnodontiformes | *Athrodon* | 1 | Ce |
| Pycnodontiformes | *Gyrodus* | 1 | Ce |
| Pycnodontiformes | *Micropycnodon* | 1 | Ce- Ca |
| Pycnodontiformes | *Phacodus* | 1 | Sa |
| Pycnodontiformes | *Polazzodus* | 1 | Sa |
| Pycnodontiformes | *Proscincetes* | 1 | Ce |
| Pycnodontiformes | *Pycnodus* | 1 | Sa–Pa |
| Scombriformes | *Sphyraenodus* | 1 | Pa |
| Scombriformes | *Cybium* | 1 | Pa |
| Scombriformes | *Mupus* | 1 | Pa |
| Semionotiformes | *Agoultichthys* | 1 | Ce |
| Semionotiformes | *Hadrodus* | 1 | Sa–Pa(?) |
| Siluriformes | *Arius* | 1 | Pa |
| Spariformes | *Nemipterus* | 1 | Pa |
| Tetraodontiformes | *Eotrigonodon* | 1 | Ma |
| Tetraodontiformes | *Ostracion* | 1 | Pa |
| Tetraodontiformes | *Stephanodus* | 3 | Ca–Ma |
| Tetraodontiformes | *Ostracion* | 1 | Ma |
| Tetraodontiformes | *Stephanodus* | 1 | Ma |
| Tselfatiiformes | *Bananogmius* | 3 | Sa–Pa(?) |
| Alepisauriformes | *Apateodus* | 1 | Sa |
| Acanthomorphata | *Sphyraena* | 1 | Ma |
| Acanthomorphata | *Acropoma* | 1 | Pa |
| Acanthomorphata | *Mene* | 1 | Pa |
| Acanthomorphata | *Pogonias* | 3 | Ce |
| Acanthopterygii | *Gigapteryx* | 1 | Ce |
| **Sarcopterygi** |  |  |  |
| Coelacanthiformes | *Macropoma* | 1 | Ce–Sa |
| Coelacanthiformes | *Mawsonia* | 1 | Ce |
| Coelacanthiformes | *Megalocoelacanthus* | 1 | Sa–Ma |

**(c). Reptiles**

| **Sauropsida** | **Genus** | **#s of species** | **Age** |
| --- | --- | --- | --- |
| Plesiosauria - Elasmosauridae | *Albertonectes* | 1 | Ca |
| Plesiosauria - Elasmosauridae | *Alzadasaurus* | 1 | Ca |
| Plesiosauria - Elasmosauridae | *Aphrosaurus* | 1 | Ma |
| Plesiosauria - Elasmosauridae | *Aristonectes* | 2 | Ca - Ma |
| Plesiosauria - Elasmosauridae | *Cimoliasaurus* | 2 | Ce - Pa |
| Plesiosauria - Elasmosauridae | *Discosaurus* | 1 | Sa |
| Plesiosauria - Elasmosauridae | *Elasmosaurus* | 7 | Sa - Ma |
| Plesiosauria - Elasmosauridae | *Fresnosaurus* | 2 | Sa - Ma |
| Plesiosauria - Elasmosauridae | *Futabasaurus* | 1 | Sa |
| Plesiosauria - Elasmosauridae | *Hydrotherosaurus* | 1 | Ma |
| Plesiosauria - Elasmosauridae | *Libonectes* | 1 | Sa |
| Plesiosauria - Elasmosauridae | *Mauisaurus* | 1 | Sa - Ma |
| Plesiosauria - Elasmosauridae? | *Morenosaurus* | 1 | Ma |
| Plesiosauria - Elasmosauridae? | *Ogmodirus* | 1 | Sa |
| Plesiosauria - Elasmosauridae | *Scanisaurus* | 1 | Ca |
| Plesiosauria - Elasmosauridae | *Styxosaurus* | 1 | Sa - Ca |
| Plesiosauria - Elasmosauridae | *Terminonatator* | 1 | Ca |
| Plesiosauria - Elasmosauridae | *Thalassomedon* | 1 | Ce |
| Plesiosauria - Elasmosauridae | *Tuarangisaurus* | 1 | Ma |
| Plesiosauria - Elasmosauridae | *Zarafasaura* | 1 | Ma |
| Plesiosauria - Polycotylidae | *Dolichorhynchops* | 2 | Sa - Ca |
| Plesiosauria - Polycotylidae | *Eopolycotylus* | 1 | Ce |
| Plesiosauria - Polycotylidae | *Georgiasaurus* | 1 | Sa |
| Plesiosauria - Polycotylidae | *Manemergus* | 1 | Sa |
| Plesiosauria - Polycotylidae | *Pahasapasaurus* | 1 | Ce |
| Plesiosauria - Polycotylidae | *Palmulasaurus* | 1 | Sa |
| Plesiosauria - Polycotylidae | *Plesiopleurodon* | 1 | Ce |
| Plesiosauria - Polycotylidae | *Polycotylus* | 1 | Sa - Ca |
| Plesiosauria - Polycotylidae | *Thililua* | 1 | Sa |
| Plesiosauria - Polycotylidae | *Trinacromerum* | 2 | Ce - Ca |
| Plesiosauria - Pliosauridae | *Brachauchenius* | 1 | Ce - Sa |
| Plesiosauria - Pliosauridae | *Megacephalosaurus* | 1 | Sa |
| Plesiosauria - Pliosauridae | *Polyptychodon* | 2 | Ce - Sa |
| Plesiosauria - Pliosauridae | *Embaphias* | 1 | Ca |
| Plesiosauria - Pliosauridae | *Taphrosaurus* | 1 | Ce |
| Mosasauroidea | *Aigialosaurus* | 2 | Ce |
| Mosasauroidea | *Carentonosaurus* | 1 | Ce |
| Mosasauroidea | *Coniasaurus* | 3 | Ce |
| Mosasauroidea | *Dolichosaurus* | 1 | Ce |
| Mosasauroidea | *Tethysaurus* | 1 | Sa |
| Mosasauridae | *Amphekepubis* | 1 | Sa |
| Mosasauridae | *Angolasaurus* | 1 | Sa - Ma |
| Mosasauridae | *Carinodens* | 2 | Ma |
| Mosasauridae | *Clidastes** | 3 | Sa - Ca |
| Mosasauridae | *Dollosaurus* | 1 | Sa - Ca |
| Mosasauridae | *Ectenosaurus* | 1 | Sa |
| Mosasauridae | *Eidolosaurus* | 1 | Ce |
| Mosasauridae | *Eonatator* | 2 | Ca |
| Mosasauridae | *Eremiasaurus* | 1 | Ma |
| Mosasauridae | *Globidens* | 3 | Ca - Ma |
| Mosasauridae | *Goronyosaurus* | 1 | Ma |
| Mosasauridae | *Hainosaurus* | 4 | Ca - Ma |
| Mosasauridae | *Halisaurus* | 4 | Sa - Ma |
| Mosasauridae | *Igdamanosaurus* | 1 | Ma |
| Mosasauridae | *Kourisodon* | 1 | Sa - Ma |
| Mosasauridae | *Latoplatecarpus* | 1 | Ca |
| Mosasauridae | *Mosasaurus* | 6 | Ca - Ma |
| Mosasauridae | *Phosphorosaurus* | 1 | Ma |
| Mosasauridae | *Platecarpus* | 4 | Sa - Ma |
| Mosasauridae | *Plioplatecarpus* | 5 | Ca - Ma |
| Mosasauridae | *Plotosaurus* | 2 | Ma |
| Mosasauridae | *Pluridens* | 1 | Ma |
| Mosasauridae | *Prognathodon* | 11 | Ca – Ma |
| Mosasauridae | *Romeosaurus* | 2 | Sa |
| Mosasauridae | *Russellosaurus* | 1 | Sa |
| Mosasauridae | *Selmasaurus* | 2 | Sa – Ca |
| Mosasauridae | *Taniwhasaurus* | 2 | Ca – Ma |
| Mosasauridae | *Tylosaurus** | 4 | Sa – Ca |
| Mosasauridae | *Yaguarasaurus* | 1 | Sa |
| Serpentes(?) | *Haasiophis* | 1 | Ce |
| Serpentes | *Pachyrhachis* | 1 | Ce |
| Testudines - Bothremydidae | *Chedighaii* | 1 | Sa – Ca |
| Testudines - Bothremydidae | *Chupacabrachelys* | 1 | Ca |
| Testudines - Bothremydidae | *Elochelys* | 1 | Ca – Ma |
| Testudines - Bothremydidae | *Foxemys* | 2 | Sa – Ma |
| Testudines - Bothremydidae | *Kurmademys* | 1 | Ma |
| Testudines - Bothremydidae | *Labrostochelys* | 1 | Pa |
| Testudines - Bothremydidae | *Nigeremys* | 2 | Ca – Ma |
| Testudines - Bothremydidae | *Polysternon* | 2 | Sa – Ma |
| Testudines - Bothremydidae | *Taphrosphys* | 3 | Ca – Pa |
| Testudines - Bothremydidae | *Acleistochelys* | 1 | Pa |
| Testudines - Bothremydidae | *Araiochelys* | 1 | Pa |
| Testudines - Bothremydidae | *Arenila* | 1 | Ma |
| Testudines - Bothremydidae | *Azabbaremys* | 1 | Pa |
| Testudines - Bothremydidae | *Bothremys* | 4 | Ca – Pa |
| Testudines - Bothremydidae | *Chedighaii* | 1 | Ca |
| Testudines - Bothremydidae | *Podocnemis* | 1 | Ce |
| Testudines - Bothremydidae | *Polysternon* | 1 | Ce & Ma(?) |
| Testudines - Bothremydidae | *Rhothonemys* | 1 | Pa |
| Testudines - Bothremydidae | *Taphrosphys* | 1 | Ca - Pa |
| Testudines - Bothremydidae | *Zolhafah* | 1 | Ma |
| Testudines - Cheloniidae | *Allopleuron* | 1 | Sa - Ca |
| Testudines - Cheloniidae | *Ctenochelys* | 3 | Sa - Ca |
| Testudines - Cheloniidae | *Dollochelys* | 1 | Pa |
| Testudines - Cheloniidae | *Gigantatypus* | 1 | Ma |
| Testudines - Cheloniidae | *Itilochelys* | 1 | Pa |
| Testudines - Cheloniidae | *Nichollsemys* | 1 | Ca |
| Testudines - Cheloniidae | *Puppigerus* | 1 | Ce |
| Testudines - Cheloniidae | *Tasbacka* | 3 | Ma - Pa |
| Testudines - Dermochelyoidae | *Corsochelys* | 1 | Ca - Ma |
| Testudines - Dermochelyoidae | *Eosphargis* | 1 | Pa |
| Testudines - Dermochelyoidae | *Mesodermochelys* | 1 | Ca - Ma |
| Testudines - Dermochelyoidae | *Ocepechelon* | 1 | Ma |
| Testudines - Durocryptodira | *Toxochelys* | 3 | Sa - Ma(?) |
| Testudines - Eucryptodira | *Borealochelys* | 1 | Sa |
| Testudines - Kinosternoidea | *Agomphus* | 3 | Sa - Ma(?) |
| Testudines - Macrobaenidae | *Osteopygis* | 6 | Ma - Pa |
| Testudines - Macrobaenidae | *Aurorachelys* | 1 | Sa |
| Testudines - Nanhsiungchelyidae | *Anomalochelys* | 1 | Ce |
| Testudines - Pancheloniidae | *Euclastes* | 3 | Ma |
| Testudines - Pancheloniidae | *Lophochelys* | 3 | Ce - Pa |
| Testudines - Pancheloniidae | *Peritresius* | 1 | Ma - Pa |
| Testudines - Pancheloniidae | *Prionochelys* | 3 | Sa |
| Testudines - Pancheloniidae(?) | *Catapleura* | 3 | Ca - Pa |
| Testudines - Panpodocnemidae | *Shweboemys* | 1 | Sa |
| Testudines - Paracryptodira | *Angolachelys* | 1 | Sa |
| Testudines - Pleurosternidae | *Glyptops* | 1 | Ce |
| Testudines - Protostegidae | *Archelon* | 1 | Sa - Ma |
| Testudines - Protostegidae | *Calcarichelys* | 1 | Ma |
| Testudines - Protostegidae | *Chelosphargis* | 1 | Sa - Ma |
| Testudines - Protostegidae | *Desmatochelys* | 1 | Sa |
| Testudines - Protostegidae | *Protostega* | 3 | Ce - Ma |
| Testudines - Protostegidae | *Rhinochelys* | 2 | Ce - Sa |
| Testudines - Protostegidae | *Teguliscapha* | 1 | Ce |
| Testudines - Protostegidae | *Terlinguachelys* | 1 | Ca |
| Testudines - Protostegidae | *Brachyopsemys* | 1 | Pa |
| Testudines - Sinemydidae | *Judithemys* | 1 | Pa |
| Testudines - Thalassemydidae | *Rhetechelys* | 1 | Pa |
| Testudines - Trionychidae | *Amyda* | 1 | Ma |
| Testudines - Trionychidae | *Aspideretoides* | 1 | Pa |
| Testudines - Trionychidae | *Aspideretoides* | 1 | Pa |
| Testudines - Trionychidae | *Hummelichelys* | 1 | Ca |
| Testudines - Toxochelyidae | *Thinochelys* | 1 | Sa - Ma |

**6. References**

1. Raymond, D. E., W. E. Osborne, C. W. Copeland, and T. L. Neathery. 1988. Alabama Stratigraphy. Geological Survey of Alabama Circular 40:1–97.

2. Jones, D. S., P. A., Mueller, J. R. Bryan, J. P. Dobson, J. E. T. Channell, J. C. Zachos, and M. A. Arthur. 1987. Biotic, geochemical, and paleomagnetic changes across the Cretaceous/Tertiary boundary at Braggs, Alabama. Geology 15:311–315.

3. Mancini, E. A. T. M. Puckett, B. H. Tew, and C. C. Smith. 1995. Upper Cretaceous sequence stratigraphy of the Mississippi – Alabama area. Gulf Coast Association of Geological Societies Transactions 45:377–384.

4. Ikejiri, T., J. Ebersole, H. L. Blewitt, and S. Ebersole. 2013. An overview of Late Cretaceous vertebrates from Alabama. Alabama Museum of Natural History Bulletin 31(1):46–71.

5. Ciampagalio, C. N., D. J. Cicimurri, J. A. Ebersole, and K. E. Runyon. 2013. A note on Late Cretaceous fish taxa recovered from stream gravels at Site AGr-43 in Greene County, Alabama. Alabama Museum of Natural History Bulletin 31-1:84–97.

6. Dunhill, A. M., M. J. Benton, R. J. Twitchett, and A. J. Newell. 2014. Testing the fossil record: Sampling proxies and scaling in the British Triassic–Jurassic. Palaeogeography, Palaeoclimatology, and Palaeoecology 404: 1–11.

7. Walker, F. M., A. M. Dunhill, M. A. Woods, A. J. Newell, and M. J. Benton. 2017. Assessing sampling of the fossil record in a geographically and stratigraphically constrained dataset: the Chalk Group of Hampshire, southern UK. Journal of the Geological Society 174: 509–521.

8. Alroy J. 2010a. Geographical, environmental and intrinsic biotic controls on Phanerozoic marine diversification. Palaeontology 53:1211–1235.

9. Mancini, E. A., B. H. Tew, and C. C. Smith. 1989. Cretaceous-Tertiary contact, Mississippi and Alabama. Journal of Foraminiferal Research 19:93–104.

10. Scotese, C. R. 2014. Atlas of Late Cretaceous Paleogeographic Maps, PALEOMAP Atlas for ArcGIS, volume 2, The Cretaceous, Maps 16 – 22, Mollweide Projection, PALEOMAP Project, Evanston, IL.

11. Carrano, M. T., J. Alroy, P. Mannion, and R. Benson. 2019. Taxonomic occurrences of Cretaceous to Paleocene Vertebrata recorded: *In*: Fossilworks, the Evolution of Terrestrial Ecosystems database, and the Paleobiology Database. <http://fossilworks.org> (January 2019).

12. Foote, M. 2000. Origination and extinction components of taxonomic diversity: general problems. Paleobiology 26 (sp4): 74–102.

13. Foote, M. & Miller, A. I. 2007. Principles of Paleontology Third Edition. W. H. Freeman and Company, 354 pp.
